# Predicting the Mechanical Properties of Super Large Aggregate Asphalt Mixture from Volumetric Parameters Using Back Propagation Neural Networks

**DOI:** 10.3390/ma19173608

**Published:** 2026-08-25

**Authors:** Xiaoping Ji, Juntao Yang, Teng Yuan, Jianyong Ma, Jie Liu, Xueyuan Zhang, Bo Wang, Chao Pu, Shiyu Zhu

**Affiliations:** 1 School of Highway, Chang’an University, Xi’an 710064, China; jixp82@163.com (X.J.); 2023021091@chd.edu.cn (J.Y.);; 2 Xinjiang Transportation Planning Survey and Design Institute Co., Ltd., Urumqi 830006, China; 3 Xinjiang Key Laboratory for Safety and Health of Transportation Infrastructure in Alpine and High-Altitude Mountainous Areas, Urumqi 830006, China; 4 Xinjiang Transportation Research Institute, Karamay 831399, China; 5 China Construction Silk Road Construction Investment Co., Xi’an 710065, China; 6 China Gezhouba Group Municipal Engineering Co., Yichang 443000, China

**Keywords:** road engineering, super large aggregate asphalt mixture, volume parameters, mechanical performance, BP neural network model

## Abstract

**Highlights:**

- Effect of aggregate particle size on the volumetric parameters of SLAM-50 is analyzed.
- Influence of volumetric parameters on properties of SLAM-50 is investigated.
- SLAM-50 performance index prediction based on Backpropagation (BP) artificial neural network.

**Abstract:**

Super-large aggregate asphalt mixtures (SLAM-50) are expected to improve rutting resistance while reducing asphalt demand, but the quantitative relationships between volumetric parameters and key performance indicators remain unclear, limiting performance-oriented mixture design. This study experimentally evaluated the volumetric properties and key performance indices of SLAM-50 across four aggregate gradations and seven asphalt contents. The measured performance indices included compressive strength, splitting strength, compressive resilient modulus, fracture energy, and dynamic stability. With increasing asphalt content, the air voids (*VV*) decreased, the voids in mineral aggregate (*VMA*) decreased initially and then increased, and the voids filled with asphalt (*VFA*) increased monotonically. All performance indices exhibited a non-monotonic trend, increasing first and then decreasing, with a balanced overall performance at 3.0–3.2% asphalt content. Linear regression models showed limited predictive capability (R^2^ = 0.5944–0.8845). To address this gap, a Backpropagation (BP) neural network was developed using asphalt content, volumetric parameters, mixture density, and gradation type as inputs, and the measured performance indices as outputs. This framework enables simultaneous multi-output prediction and captures the nonlinear, coupled relationships among variables. The model achieved R^2^ > 0.91 for both training and testing datasets, demonstrating its ability to accurately predict SLAM-50 performance. These findings provide a practical, data-driven basis for performance-oriented mixture design and optimization, addressing the current scientific gap and offering guidance for engineering practice.

## 1. Introduction

With the continuous increase in traffic volume and the widespread adoption of heavy-duty vehicles, modern pavement structures are placing increasingly stringent demands on the strength, stability, and durability of asphalt mixtures. Large-aggregate asphalt mixtures, such as asphalt treated base-30 (named ATB-30, maximum nominal particle size = 30 mm) and asphalt treated base-40 (named ATB-40, maximum nominal particle size = 40 mm), are widely used in high-grade highway pavement structures due to their strong interlocking skeletons, high load-bearing capacity, excellent moisture resistance, and reduced asphalt content [1,2,3,4]. With advancements in paving technology and compaction methods, construction quality control for large-aggregate mixtures has significantly improved, enabling the practical application of super large aggregates in asphalt mixtures. Against this backdrop, super large aggregate mixture (named SLAM-50, maximum nominal particle size = 50 mm) has been proposed for use in pavement engineering. Previous studies have shown that the incorporation of super large aggregates can form a stable interlocked skeleton, significantly enhancing the mechanical performance, fatigue resistance, and rutting resistance of asphalt mixtures. Furthermore, the use of larger aggregates reduces the required asphalt content and pavement thickness, lowering both material consumption and construction costs. These advantages highlight the considerable application potential and research value of SLAM-50 in high-performance pavement structures [5,6].

Proper design of SLAM-50 asphalt mixtures is essential to realizing their full structural advantages and performance potential. Currently, the primary mix design methods include the Marshall method [7], Superpave method [8,9], and Vibration Compaction Method [10]. Among these, the Marshall compaction method (MCM) is the most widely used, particularly in low-volume roads. However, as traffic demand increases, the limitations of MCM under heavy traffic conditions have become more pronounced. To better simulate field compaction, the Vibration Compaction Method (VVCM) has been developed as a more realistic laboratory compaction method that mimics dynamic loading conditions [11,12,13,14]. Xue [15] compared SMA-13 mixtures prepared using MCM and VVCM, finding that VVCM specimens exhibited superior penetration shear strength, tensile strength, post-moisture conditioning shear strength, and rutting resistance—closer to field performance. Moreover, the volumetric properties and performance of VVCM-compacted specimens showed strong agreement with field cores.

Although these compaction methods differ primarily in their equipment and procedures, the performance differences observed among the resulting asphalt mixture specimens are fundamentally attributed to the variations in volumetric parameters generated during compaction [16,17,18]. Therefore, understanding how volumetric parameters such as air voids (*VV*), voids in mineral aggregate (*VMA*), and voids filled with asphalt (*VFA*) affect asphalt mixture performance is essential for optimizing mix design across compaction methods. *VV* refers to the percentage of voids not filled by binder or aggregate in the compacted mixture and directly influences high-temperature stability, moisture susceptibility, and fatigue life [19,20,21]. Excessive *VV* can result in reduced structural stability and fatigue life of the asphalt mixture, while overly low *VV* may impair rutting resistance [22,23,24,25,26]. *VMA* represents the volume of intergranular voids within the mineral aggregate framework, including air and asphalt, and reflects the space available for binder. Inadequate *VMA* may cause insufficient binder film thickness and aggregate stripping, whereas overly high *VMA* can increase costs and reduce fatigue resistance. Rational *VMA* design helps ensure proper film thickness and long-term performance [27,28,29]. *VFA*, defined as the percentage of *VMA* filled by binder, affects water damage resistance, cracking, and rutting behavior. *VFA* values below 65% may lead to insufficient coating and increased permeability, while excessive *VFA* may cause bleeding and saturation. Studies suggest optimal performance is achieved when *VFA* is controlled between 65% and 80% [11,30].

Given the essential role of volumetric parameters—*VV*, *VMA*, and *VFA*—in determining the mechanical strength, moisture resistance, and long-term durability of asphalt mixtures, considerable attention has been devoted to their optimization in recent years. A growing body of research has focused on establishing scientifically based volumetric design ranges that align with performance targets, aiming to improve the structural integrity and serviceability of asphalt pavements under various traffic and environmental conditions. Jiang et al. [11] investigated the influence of volumetric parameters on the mechanical properties of ATB-30 asphalt mixtures prepared using the Vertical Vibration Compaction Method (VVCM). The results indicated that *VV* and *VFA* were strongly correlated with mechanical performance, while *VMA* exhibited relatively weak correlation. Based on these findings, the volumetric parameter ranges specifically tailored for ATB-30 mixtures were proposed. Zhang et al. [31] investigated the performance evolution mechanisms of AC-16 hot mix asphalt using the Uniaxial Penetration Shear Test (UPST), Low-Temperature Bending Test (LTBT), and Four-Point Bending Test (4PBT). Through these tests, they identified the key volumetric parameters that significantly influence mixture performance and subsequently proposed corresponding volumetric design criteria to enhance the mixture’s structural and performance. Zhang [32] conducted a comprehensive field study using data from 71 pavement sections across 24 test roads located in four major climatic regions of the United States. Their analysis focused on the sensitivity of rutting and cracking performance to variations in volumetric parameters. The results revealed that lower *VFA* values were strongly associated with top-down longitudinal cracking. Moreover, *VMA* exhibited a non-linear influence on rutting depth, suggesting the existence of a sensitive range in which small changes in *VMA* significantly affected rutting resistance. Al Kaaf [33] compared the volumetric properties of asphalt mixtures with a nominal maximum aggregate size of 25 mm designed using the Marshall and Superpave methods. The results indicated that Marshall-compacted specimens exhibited lower *VMA* and *VFA* values compared to those designed with the Superpave method, reflecting relatively lower structural compactness. Kadhim et al. [34] investigated the effects of incorporating varying proportions of walnut shell ash and limestone into AC-20 mixtures on volumetric parameters such as *VMA* and *VV*, as well as on performance indicators including Marshall stability and flow value. Based on the results, they identified optimal volumetric parameter ranges corresponding to improved mechanical properties. Aladegboye [35] applied response surface methodology to investigate the effects of coarse aggregate, fine aggregate, filler, binder content, and cassava peel ash on volumetric and Marshall performance indicators, and developed volumetric parameter predictive models for performance optimization.

Although prior studies have demonstrated that volumetric properties (e.g., air void content, voids in mineral aggregate, and voids filled with asphalt) are key indicators for characterizing asphalt mixture performance, the relationships between these volumetric properties and mechanical responses may exhibit nonlinear and coupled characteristics. Previous studies mainly relied on correlation analysis, linear or quadratic regression, or response surface methodology to establish these relationships. While these approaches can effectively identify trends for individual performance indicators under limited experimental conditions, they may be insufficient to fully capture the interactions among multiple volumetric parameters, especially for super-large aggregate asphalt mixtures and multiple performance metrics. Linear regression models remain useful for trend identification due to their simplicity, interpretability, and robustness. However, more flexible nonlinear approaches, such as neural networks and deep learning, may provide additional capability for modeling complex input–output relationships. Consequently, data-driven methods with nonlinear approximation capacity have emerged as a promising complementary approach for predicting asphalt mixture performance. Leveraging their self-learning and nonlinear fitting capabilities, neural networks and deep learning have demonstrated improved robustness and predictive accuracy in civil engineering materials modeling [36,37].

Existing research has verified the effectiveness of intelligent algorithms for predicting various asphalt mixture performance indicators. Niu et al. developed a Backpropagation (Back Propagation, BP) neural network model that used mesoscopic parameters as inputs and mechanical performance as outputs; the correlation coefficient between predicted and measured values exceeded 0.93, indicating a satisfactory predictive capability [38]. Rahman et al. applied machine learning to estimate rut depth and tensile strength using aggregate gradation and water absorption, asphalt content, Performance Grade (PG) and the number of wheel load applications as model inputs; their results suggested reliable prediction of mixture performance [39]. Shafabakhsh et al. investigated the application of an Artificial Neural Network (ANN) for permanent deformation prediction of nano-additive–modified asphalt mixtures, incorporating aggregate source, additive type and dosage, temperature, and stress as inputs and mixture strain as the output; they reported that an ANN with 10 neurons in the hidden layer provided accurate prediction of the final strain [40]. Barugahare et al. established an ANN-based dynamic modulus prediction model and validated it using experimental data from asphalt mixtures in South Carolina, showing that the ANN model outperformed regression-based methods and could still achieve high accuracy with a reduced set of input variables [41]. Tian et al. employed a neural-network framework for fatigue life prediction using temperature, stress level, stress, and flexural strength as inputs, and reported a prediction error of only 4.7% [5]. Beyond neural networks, Majidifard et al. proposed a rut depth prediction model based on Gene Expression Programming (GEP) using PG, mixture type, aggregate size and gradation, and asphalt content as inputs [42]. Liu et al. further compared multiple linear regression, Generalized Regression Neural Network (GRNN), and Support Vector Machine (SVM) approaches under small-sample conditions to develop a dynamic modulus prediction model for high-modulus asphalt mixtures [43].

While neural networks, deep learning, and related data-driven methods have been extensively reported for performance prediction of conventional asphalt mixtures, studies focusing on large-aggregate asphalt mixtures (i.e., mixtures with a very large nominal maximum aggregate size) remain relatively scarce. Specifically, there is a lack of systematic studies on the relationships between volumetric parameters and mechanical performance for super-large aggregate asphalt mixtures, and the application of neural networks to model these multi-variable, coupled relationships is limited. In particular, systematic investigations that integrate comprehensive performance testing with neural-network-based predictive modeling for large-stone asphalt mixtures are still limited, which hinders efficient mixture design optimization and rapid performance-oriented decision-making in engineering practice.

**Figure 1:**
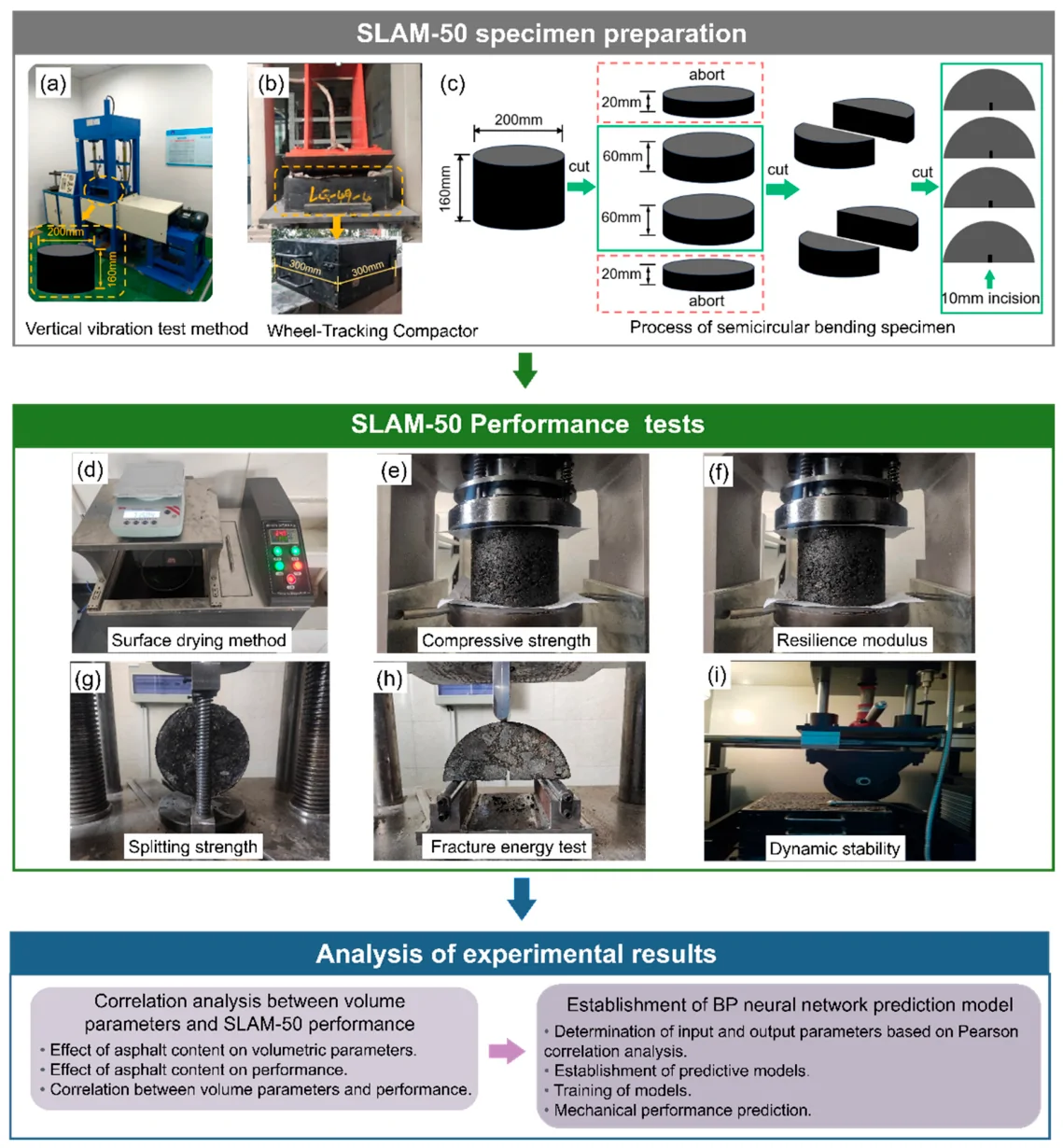
Research flowchart of this study (**a**) Vertical vibration test method; (**b**) Wheel-Tracking Compactor; (**c**) Process of semicircular bending specimen; (**d**) Surface drying method; (**e**) Compressive strength; (**f**) Resilience modulus; (**g**) Splitting strength; (**h**) Fracture energy test; (**i**) Dynamic stability.

In this study, SLAM-50 specimens with varying aggregate gradations and asphalt contents were fabricated using a vertical vibratory compactor and a wheel tracking compactor. First, the key density-related indices were determined using the maximum theoretical relative density test and the saturated surface-dry (SSD) method, and the volumetric properties of SLAM-50 were subsequently calculated. Next, several laboratory tests were conducted to characterize the performance of SLAM-50: uniaxial compression testing was used to quantify the compressive strength, splitting strength, and compressive resilient modulus; the semi-circular bending (SCB) test was performed to evaluate cracking resistance through fracture energy; and a wheel-tracking rutting test was employed to determine the dynamic stability. Furthermore, the correlations between the measured mechanical properties and the corresponding volumetric parameters were investigated to clarify the role of volumetric characteristics in governing the performance of SLAM-50. Finally, asphalt content, volumetric parameters, density and gradation type were taken as model inputs and the measured performance indices as outputs to develop a BP neural-network-based prediction model for SLAM-50 performance. The research framework is summarized in Figure 1. The outcomes of this work are expected to provide a practical basis for mixture design and construction of SLAM-50.

## 2. Objectives

This study aims to elucidate the correlations between volumetric properties and performance characteristics of super large aggregate asphalt mixtures. The specific objectives are as follows:

(1) To investigate the effects of aggregate gradation and asphalt content on the volumetric properties of SLAM-50.
(2) To quantify the variation trends of SLAM-50 performance indices under different volumetric conditions.
(3) To develop a BP neural-network-based prediction model for SLAM-50 performance.

## 3. Experiment Programs

### 3.1. Materials

#### 3.1.1. Raw Materials

In this study, Karamay 90# base asphalt (KMMY, Karamay Petrochemical Company, Karamay, China) was used as the matrix asphalt. Matrix asphalt refers to the binder phase that surrounds and binds aggregates and fine particles, forming the continuous phase responsible for transmitting stresses within SLAM-50. Its primary technical indicators are presented in Table 1 in accordance with the Standard Test Methods of Bitumen and Bituminous Mixtures for Highway Engineering (JTG 3410-2025) [10], and the test results meet the specification requirements.

The basalt crushed stone aggregate used was obtained from Baiyanghe Sand and Gravel Quarry (Urumqi, China). They were divided into different particle sizes by an electric vibrating sieve machine and divided into coarse aggregate and fine aggregate with the boundary of 2.36 mm. The basic properties of the aggregates were determined according to the “Test methods of aggregate for highway engineering” (JTG E42-2005) [44]. The main technical indices are shown in Table 2 and Table 3, indicating the test results could meet the specified requirements.

The employed mineral powder is limestone mineral powder, whose basic performances are evaluated through the Test Methods for Aggregates in Highway Engineering [44]. The key technical indicators are presented in Table 4, and all test results comply with the regulatory requirements.

#### 3.1.2. Gradation Design

**Figure 2:**
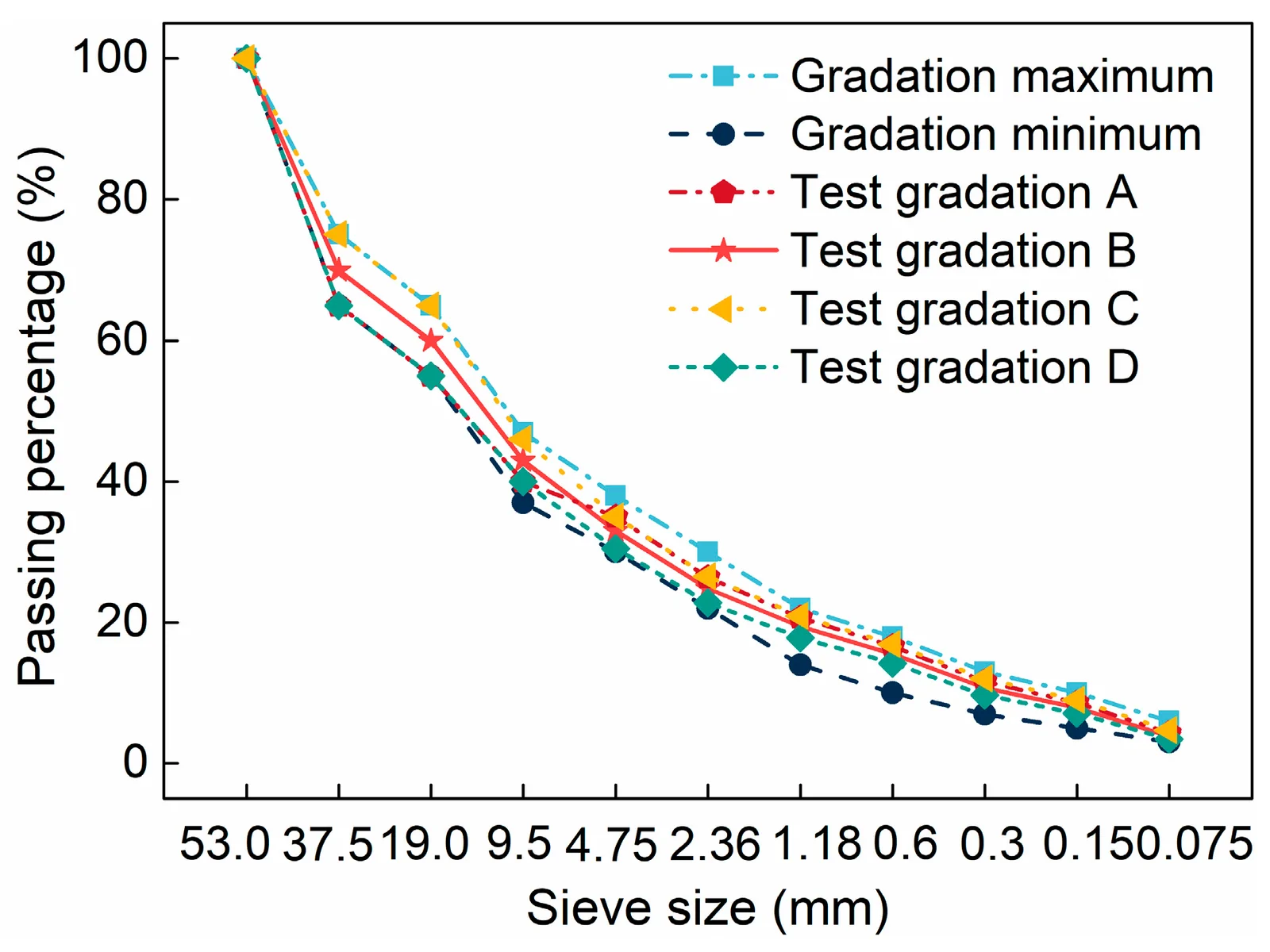
Gradation curve of SLAM-50.

Based on previous studies on SLAM-50 [45,46], four SLAM-50 gradation types were selected for this study: S-shaped gradation (A), median gradation (B), fine-aggregate-rich gradation (C), and coarse-aggregate-rich gradation (D), as illustrated in Figure 2. To improve clarity and facilitate comparison among Gradations A–D, a table presenting the aggregate compositions at each sieve size has been added following Figure 2. The four gradations were selected to investigate the influence of aggregate size distribution on the volumetric characteristics and performance of SLAM-50. Gradation A is an S-shaped gradation designed to achieve a balanced aggregate skeleton structure. Gradation B serves as an intermediate gradation and reference mixture. Gradation C contains a relatively higher proportion of fine aggregates, which is expected to increase the contribution of asphalt mastic while reducing aggregate interlock. In contrast, Gradation D contains a relatively higher proportion of coarse aggregates and is expected to enhance stone-on-stone contact and skeleton stability. These gradation characteristics provide a mechanistic basis for interpreting the differences observed in volumetric parameters and performance indices in subsequent sections.

The optimal asphalt contents (OAC) for each gradation, determined through the VVCM [10], were 3.0%, 3.1%, 3.3% and 3.0%, respectively. The OAC values of the four gradations were determined based on the combined consideration of volumetric properties and performance responses obtained from the preliminary mixture design [45,46]. For each gradation, a series of asphalt contents centered around the preliminary OAC was evaluated, as listed in Table 5. The final OAC was selected as the asphalt content at which the mixture exhibited a balanced volumetric structure, including appropriate *VV*, *VMA*, and *VFA* values, together with satisfactory strength, stiffness, cracking resistance, and rutting resistance. This procedure was adopted because SLAM-50 contains a larger nominal maximum aggregate size than conventional asphalt mixtures, and its mixture design should account for both aggregate skeleton stability and asphalt mastic filling. To expand the sample set, five asphalt content levels (OAC, OAC ± 0.2%, OAC ± 0.4%, OAC ± 0.6%) was designed for each gradation (A, B, C, D-gradation), as detailed in Table 6.

It should be noted that the optimum asphalt contents of Gradations A–D are relatively close, ranging from 3.0% to 3.3%. This is mainly attributed to the fact that all mixtures belong to the SLAM-50 system and possess the same nominal maximum aggregate size of 53 mm. The aggregate skeleton is primarily controlled by coarse aggregates, resulting in relatively small variations in overall aggregate specific surface area among the four gradations. Consequently, the asphalt demand required for aggregate coating and void filling remains within a narrow range. Although the gradations differ in the proportions of coarse and fine aggregates, these variations mainly affect aggregate interlock and skeleton structure rather than causing substantial changes in asphalt demand.

#### 3.1.3. Specimen Preparation

Figure 1a illustrates the specimen preparation methods employed in this study for asphalt mixtures. Prior to compaction, the aggregates and asphalt binder were heated separately to 160–165 °C, and the mixed materials were maintained at the compaction temperature before specimen fabrication. Cylindrical SLAM-50 specimens, with a diameter of 200 mm and a height of 160 mm, were fabricated using the Vertical Vibratory Compaction method (VVTE) to simulate heavy traffic conditions. During compaction, the VVTE applies a vertical force to rearrange and densify the mixture particles, enhancing compactness and mechanical properties. The upper and lower components of the VVTE weigh 122 kg and 180 kg, respectively, with a working frequency of 40 Hz, a compaction duration of 90 s, and a rated amplitude of 1.2 mm [45]. These cylindrical specimens were subsequently used for strength testing or trimmed to the desired shape and thickness for other evaluations. Additionally, rutting slab specimens measuring 300 mm × 300 mm × 100 mm were prepared using the wheel-tracking compaction method. For each testing condition and gradation, six replicate specimens were prepared for all tests, and the mean values were used in subsequent analysis.

### 3.2. Test Methods

Figure 1d–f provides the testing flowchart used in this study. The evaluation of asphalt mixture specimens involved tests of volumetric parameters, along with mechanical and road performance assessments. A detailed description of the testing procedure is provided below.

#### 3.2.1. Volume Parameters

The volumetric parameters of SLAM-50 were determined through the following procedure. First, loose mixture samples were prepared at the designed asphalt contents and aggregate gradations, and the theoretical maximum relative density of each mixture was measured in accordance with the standard test method. Second, compacted cylindrical specimens were prepared using the vertical vibratory compaction method. After cooling to room temperature, the surface-dry mass, submerged mass, and dry mass of each compacted specimen were measured to determine the bulk relative density using the saturated surface-dry method. Third, based on the measured theoretical maximum relative density, bulk relative density, asphalt content, and aggregate relative density, the volumetric parameters, including air voids (*VV*), voids in mineral aggregate (*VMA*), and voids filled with asphalt (*VFA*), were calculated using Equations (1)–(3). For each gradation and asphalt content, the volumetric parameters were calculated from replicate specimens, and the average values were used for subsequent analysis. Specimens with obvious surface defects, segregation, or abnormal density values were excluded and remade to ensure the reliability of the volumetric measurements. In accordance with the surface drying method (as shown in Figure 1d) specified in Standard Test Methods of Bitumen and Bituminous Mixtures for Highway Engineering (T0705-2025) [10], the values of *VV*, *VMA* and *VFA* can be determined using Equation (1), Equation (2) and Equation (3), respectively.

(1) $$VV=(1-\frac{\gamma_{f}}{\gamma_{t}})\times 100$$

(2) $$VMA=(1-\frac{\gamma_{f}}{\gamma_{sb}}\times \frac{P_{s}}{100})\times 100$$

(3) $$VFA=\frac{VMA-VV}{VMA}$$

where, $P_{s}$ = aggregate, percent by total weight of mix (%); $\gamma_{sb}$ = bulk specific gravity of the aggregate; $\gamma_{f}$ = bulk specific gravity of the mixture; $\gamma_{t}$ = theoretical maximum density.

#### 3.2.2. Performance Test

In this section, several tests for SLAM-50 have been conducted to deal with the mechanical performances under regular and special environmental conditions during road service, including compressive strength, low temperatures fracture, splitting strengths under ambient temperature and freeze–thaw condition. Compressive strength, splitting strength, and compressive resilient modulus were selected to characterize the mechanical behavior of the mixtures. Fracture energy obtained from the SCB test was used to evaluate cracking resistance, whereas dynamic stability obtained from the wheel-tracking test was adopted to characterize high-temperature rutting resistance.

##### Compressive Strength

The uniaxial compression test was conducted to evaluate the compressive strength of SLAM-50 using the cylindrical specimens prepared as described in Section 3.1.3, in accordance with the *Standard Test Methods of Bitumen and Bituminous Mixtures for Highway Engineering* (T0705-2025) [10]. The experimental setup is shown in Figure 1e. During testing, a vertical load was applied to the specimens at a constant rate of 2 mm/min. The compressive strength can be calculated using Equation (10) and test results.

(4) $$\sigma_{c}=\frac{4P}{\pi d^{2}}$$

where, $\sigma_{c}$ = the compressive strength (MPa); P = the maximum load (N); d = diameter of the specimen (mm).

##### Splitting Strength

The splitting strength of the SLAM-50 prepared in Section 3.1.3 was characterized using the splitting strength in accordance with the *Standard Test Methods of Bitumen and Bituminous Mixtures for Highway Engineering* (T0705-2025) [10]. Given the large size of the SLAM-50 asphalt mixture specimen, a customized compression strip measuring 200 mm in length, 25.4 mm in width, and featuring an inner radius of curvature of 100 mm was employed for the splitting test. Figure 1g illustrates the test device used for the splitting strength test. During the experiment, the cylindrical specimens were first cooled to 20 °C in the laboratory, after which a vertical load was added to the specimen at a constant rate of 50 mm/min. The splitting strength can be obtained in accordance with Equation (11) as well as test results.

(5) $$\sigma_{t}=\frac{2P}{\pi ah}(\sin2\alpha -\frac{a}{d})$$

in which, $\sigma_{t}$ = the splitting strength (MPa); P = the maximum load (N); a = the widths of compression strip (mm); h = the height of the specimen (mm); α = half of the angle corresponding to the arc length of the pressure bar.

##### Compressive Resilience Modulus

According to *Standard Test Methods of Bitumen and Bituminous Mixtures for Highway Engineering* (T0705-2025) [10], the cylindrical specimens prepared in Section 3.1.3 were tested to determine the uniaxial compressive resilient modulus, as shown in Figure 1f. During the test, a multi-stage loading protocol was adopted, with the applied load set to 0.1*P*, 0.2*P*, 0.3*P*, 0.4*P*, 0.5*P*, 0.6*P*, and 0.7*P* (where *P* denotes the maximum load specified in the standard). The first load level (0.1*P*) was applied at a constant loading rate of 2 mm/min, and the dial gauge reading together with the actual applied load was recorded immediately. The specimen was then unloaded to zero at the same rate, and the rebound deformation after 30 s, denoted as Δ*L*_1_, was measured using the dial gauge. The same procedure was repeated for load levels 2–7 to obtain the rebound deformation Δ*L_i_* corresponding to each load level.

For each stage, the actual compressive stress sustained by the specimen, *q*_i_, was calculated using Equation (6). A *q*_i_–Δ*L_i_* relationship was then plotted with *q*_i_ as the abscissa and Δ*L*_i_ as the ordinate. By extending the fitted relationship to intersect the coordinate axes, a corrected origin was determined to account for seating and system compliance. Using the corrected origin as the reference point, the compressive stress and the corresponding rebound deformation at the fifth load level (0.5σ_c_)—i.e., *q*_5_ and ΔL_5_—were extracted. Finally, the compressive resilient modulus of the asphalt mixture was calculated according to Equation (7).

(6) $$q_{i}=\frac{4P_{i}}{\pi d^{2}}$$

(7) $$E_{c}=\frac{q_{5}\times h}{\Delta L_{5}}$$

where *q*_i_ is the compressive stress corresponding to the applied load *P_i_* at the *i*-th loading stage (MPa); *P_i_* is the applied load at the *i*-th stage (N); *E*_c_ is the compressive resilient modulus (MPa); *q*_5_ is the compressive stress corresponding to the fifth loading stage (MPa); *h* is the specimen height (mm); and Δ*L*_5_ is the rebound deformation at the fifth loading stage after origin correction (mm).

##### Low-Temperature Property

The low-temperature property of SLAM-50 asphalt mixtures was characterized using the low temperature fracture test (LTFT) in accordance with *AASHTO Designation: TP 105-13* [47]. The LTFT experimental setup is also shown in Figure 1h. In the test, the SLAM-50 cylindrical specimens prepared in Section 3.1.3 were first cut into a semicircle shape with a radius of 100 mm and a thickness of 60 mm, and a 20 mm notch was cut at the bottom center of the semicircular specimen, as depicted in Figure 1h. The prepared specimens were then stored at −10 °C for at least 5 h. Finally, a vertical load was applied to the semicircular specimens at a constant rate of 5 mm/min. The fracture energy (G*_f_*) was calculated in accordance with Equation (8) to describe the low-temperature performance of the mixture. A higher value of G*_f_* indicates improved resistance to low-temperature cracking of the mixture.

(8) $$G_{f}=\frac{W_{f}}{(r-a)t}$$

where, *W_f_* = the fracture energy (J); *r* = the radius of specimens (kN); *a* = the length of slit at the center of the specimen circle (as is 10 mm); *t* = the thickness of the specimen (mm).

##### High-Temperature Property

In accordance with *Standard Test Methods of Bitumen and Bituminous Mixtures for Highway Engineering* (T0719-2025) [10], the wheel-tracking test was employed to evaluate the high-temperature performance of SLAM-50. Rutting slab specimens with dimensions of 300 mm × 300 mm × 100 mm were first fabricated using the wheel-tracking compaction method, as illustrated in Figure 1i. After demolding, all specimens were conditioned in a temperature-controlled chamber at 60 ± 1 °C for 7 h to ensure thermal equilibrium.

The dynamic stability (*D*_S_) of the asphalt mixture was subsequently determined using a wheel-tracking testing apparatus. During testing, a rubber wheel repeatedly traversed the specimen surface in a dry environment at 60 ± 1 °C, with a reciprocating frequency of 42 ± 1 passes per minute and a contact pressure of 0.7 ± 0.05 MPa. The wheel-tracking system automatically recorded the rut depth as a function of loading time and number of wheel passes. Dynamic stability was calculated from the slope of the rutting curve within the time interval of 45–60 min, as expressed in Equation (9):

(9) $$D_{s}=\frac{N_{2}-N_{1}}{d_{2}-d_{1}}$$

where *N*_1_ and *N*_2_ represent the number of wheel passes at 45 and 60 min, respectively (times), and *d*_1_ and *d*_1_ denote the corresponding rut depths (mm).

### 3.3. Backpropagation Neural Network

Artificial Neural Networks (Artificial Neural Network, ANN) are nonlinear mapping models composed of a large number of interconnected neurons with weighted connections, and are widely used to approximate complex input–output relationships [48]. A typical ANN consists of an input layer, one or more hidden layers, and an output layer [49], in which adjacent layers are connected through weights and biases. Nonlinearity is introduced through activation functions, such as Sigmoid, hyperbolic tangent (tanh), and Rectified Linear Unit (ReLU).

The training process of an ANN aims to minimize a predefined loss function using sample data, during which the network weights and biases are iteratively updated through error backpropagation and gradient-based optimization algorithms, enabling the network output to gradually converge toward the target values [50]. Among various ANN architectures, feedforward neural networks trained using the BP algorithm—commonly referred to as BP neural networks—have been increasingly applied to asphalt mixture and pavement performance prediction due to their strong capability in handling multivariable coupling and highly nonlinear engineering problems [51,52].

Accordingly, a BP neural network was adopted in this study to establish a multi-performance prediction model for SLAM-50. The asphalt content (AC), volumetric parameters, density of SLAM-50 ($\rho_{f}$) and gradation type (GT) of SLAM-50 were selected as input variables, while the mechanical performance indices, dynamic stability, and fracture energy were taken as output variables. The proposed model aims to capture the intrinsic mapping relationships between volumetric parameters and performance responses of super-large aggregate asphalt mixtures.

#### 3.3.1. Model Error Analysis

To eliminate the influence of differences in parameter magnitudes and measurement units on model training and analysis, all input and output variables were first normalized using Equation (10), scaling the data into the range of [0, 1].

(10) $$\left\{\begin{array}{l} {\overset{⌢}{x}}_{i}=\frac{x_{i}-\min(x_{i})}{\max(x_{i})-\min(x_{i})}\\ {\overset{⌢}{y}}_{i}=\frac{y_{i}-\min(y_{i})}{\max(y_{i})-\min(y_{i})} \end{array}\right.$$

where, ${\overset{⌢}{x}}_{i}$, ${\overset{⌢}{y}}_{i}$ denotes the normalized variable, $x_{i}$, $y_{i}$ is the original value, and $\min(x_{i})$, $\min(y_{i})$ and $\max(x_{i})$, $\max(y_{i})$ represent the minimum and maximum values of the corresponding variable, respectively.

The experimental dataset was then used to train the BP neural network, while the validation dataset obtained after training was employed to evaluate the predictive performance of the model. The coefficient of determination (*R*^2^), mean absolute error (MAE), and root mean square error (RMSE) were selected as evaluation metrics to quantify model accuracy. These statistical indicators were calculated using Equations (11)–(13):

(11) $$R^{2}=\frac{(\sum_{i=1}^{n}\left(a_{i}-{\bar{a}}_{i})(p_{i}-p_{i}\right){)}^{2}}{\sum_{i=1}^{n}{\left(a_{i}-{\bar{a}}_{i}\right)}^{2}\sum_{i=1}^{n}{\left(p_{i}-p_{i}\right)}^{2}}$$

(12) $$MAE=\frac{1}{n}\sum_{i=1}^{n}\left|a_{i}-p_{i}\right|$$

(13) $$RMSE=\sqrt{\frac{\sum_{i=1}^{n}{\left(a_{i}-p_{i}\right)}^{2}}{n}}$$

in which, $a_{i}$ denote the experimental (measured) and $p_{i}$ predicted values of the *i*-th sample, respectively, and *n* is the total number of samples.

#### 3.3.2. Activation Function

Activation functions are introduced in neural networks to incorporate nonlinearity and thereby enhance the network’s capability to approximate complex relationships. Although the Sigmoid function has been widely used in conventional neural-network models, it is prone to gradient saturation, which may lead to vanishing gradients and slow convergence. Previous studies [53] have reported that, compared with S-shaped activation functions, the rectified linear unit (ReLU) can achieve comparable mapping performance with fewer training samples, while offering improved convergence behavior. Accordingly, ReLU was adopted as the activation function in the hidden layer in this study to improve training efficiency and numerical stability, whereas an S-shaped (Sigmoid) activation function was employed in the output layer to constrain the predicted values to the range of [0, 1]. The mathematical expressions of the ReLU and Sigmoid activation functions are given in Equations (14) and (15):

(14) $$f(x)=\max(x,0)=\left\{\begin{array}{l} x,x>0\\ 0,x<0 \end{array}\right.$$

(15) $$g(x)=\frac{1}{1+e^{-x}}$$

## 4. Results and Discussion

### 4.1. Volume Parameters

**Figure 3:**
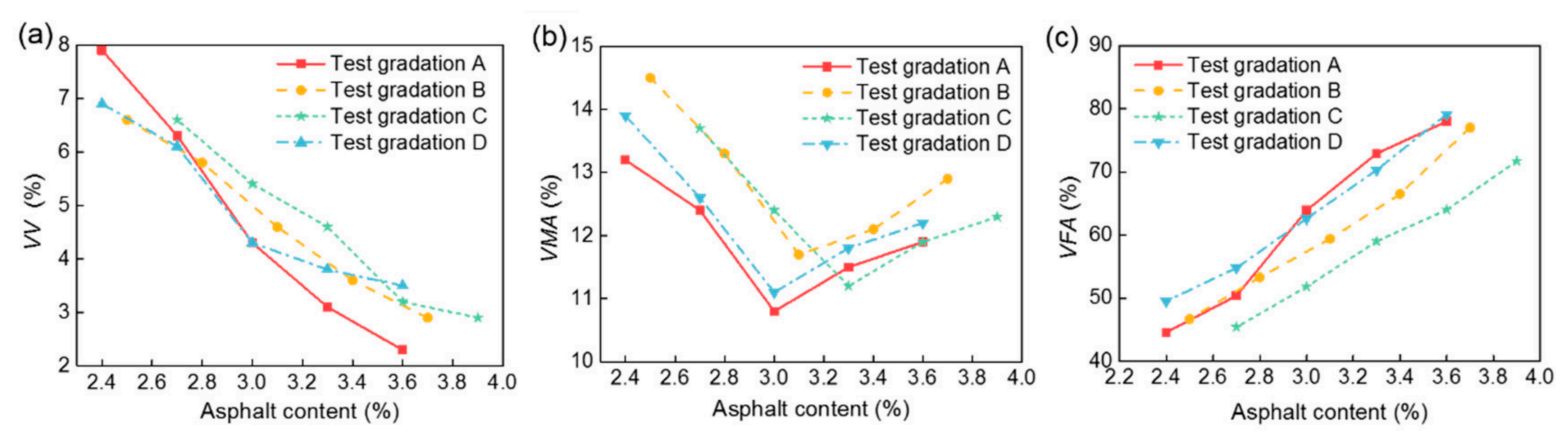
Variation curves of volume parameters with asphalt content: (**a**) *VV* versus asphalt content. (**b**) *VMA* versus asphalt content. (**c**) *VFA* versus asphalt content.

Figure 3 shows the variations of *VV*, *VFA*, and *VMA* with asphalt content for SLAM-50 prepared using four aggregate gradations. Consistent trends were observed across all gradations. Specifically, *VFA* increased with increasing asphalt content, whereas *VV* and *VMA* generally decreased. Notably, when asphalt content exceeded a critical level, *VMA* exhibited a turning point and began to increase. This non-monotonic response can be explained by the competition between densification and skeleton dilation mechanisms. At relatively low asphalt contents, additional binder improves coating and lubrication at aggregate contacts, reduces internal friction, and facilitates compaction, leading to higher *VFA* and lower *VV* and *VMA*. As asphalt content continues to increase, excess binder tends to reduce stone-on-stone contact and weaken aggregate interlock, which may induce dilation of the aggregate skeleton and consequently increase *VMA*. It is also observed that, at the same asphalt content, Gradations A and D exhibited lower VV and higher *VFA* compared with the other gradations. This behavior can be attributed to their aggregate gradation characteristics. Gradation D contains a relatively higher proportion of coarse aggregates, which facilitates stone-on-stone contact and skeleton formation. In contrast, the S-shaped structure of Gradation A enables more efficient particle packing and intergranular void filling across multiple aggregate sizes. Consequently, both gradations exhibit lower air void contents and higher asphalt-filled void ratios than the other mixtures. In comparison, Gradation C contains a higher proportion of fine aggregates, which increases dependence on asphalt mastic and reduces aggregate interlock, resulting in higher VV and lower *VFA*.

### 4.2. Mechanical Properties

**Figure 4:**
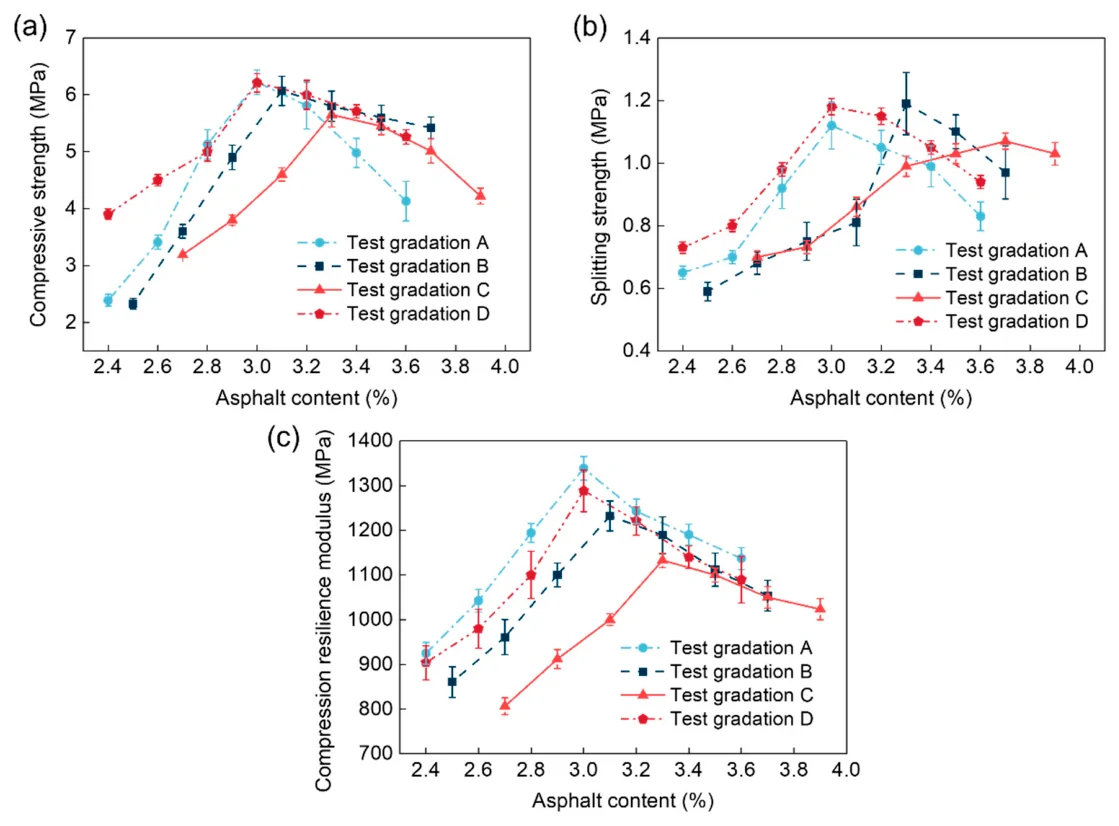
SLAM-50 performance test results: (**a**) Compressive strength. (**b**) Splitting strength. (**c**) Compression resistance resilience modulus.

Figure 4 presents the variation of strength-related indices of SLAM-50 with asphalt content for the four gradations. As shown, the strengths of all mixtures increased progressively with increasing asphalt content and reached peak values when the asphalt content was close to 3.1%, after which the compressive strength decreased. For compressive strength, Gradation A reached a maximum of 6.22 MPa at 3.0% asphalt content, Gradation B peaked at 6.07 MPa at 3.1%, Gradation C peaked at 5.65 MPa at 3.3%, and Gradation D reached 6.21 MPa at 3.0%. A similar trend was observed for splitting strength. Specifically, the splitting strength increased from approximately 0.59–0.73 MPa at asphalt contents of 2.4–2.7% to its maximum at higher asphalt contents (3.0–3.4%): the peak values were 1.12 MPa at 3.0% for Gradation A, 1.19 MPa at 3.3% for Gradation B, 1.07 MPa at 3.7% for Gradation C, and 1.18 MPa at 3.0% for Gradation D, followed by a slight decline. The compressive resilient modulus exhibited the same non-monotonic pattern, increasing first and then decreasing, with peak modulus occurring at asphalt contents of 3.0% (Gradation A), 3.1% (Gradation B), 3.3% (Gradation C), and 3.0% (Gradation D).

Compared with Gradations A, B, and D, mixtures with Gradation C exhibited lower strength and stiffness and required a higher asphalt content to reach peak strength and modulus. This behavior is likely attributable to the higher fine aggregate content and reduced coarse aggregate fraction in Gradation C, which weakens stone-on-stone contact and compromises the stability of the load-bearing aggregate skeleton. Consequently, the mechanical response becomes more dependent on the asphalt mastic than on a well-interlocked coarse aggregate framework, resulting in reduced mixture stiffness and strength. This non-monotonic variation can be explained by the combined effects of asphalt coating, aggregate bonding, and skeleton stability. At relatively low asphalt contents, insufficient binder limits aggregate coating and stress transfer, resulting in lower strength and stiffness. With increasing asphalt content, improved binder coating and aggregate bonding enhance the load-bearing capacity of the mixture. However, excessive asphalt content weakens stone-on-stone contact and increases the dependence on asphalt mastic, leading to reductions in compressive strength, splitting strength, and resilient modulus. Similar effects of volumetric parameters and asphalt content on asphalt mixture mechanical properties have been reported in previous studies [11,31].

### 4.3. Fracture Energy Results

**Figure 5:**
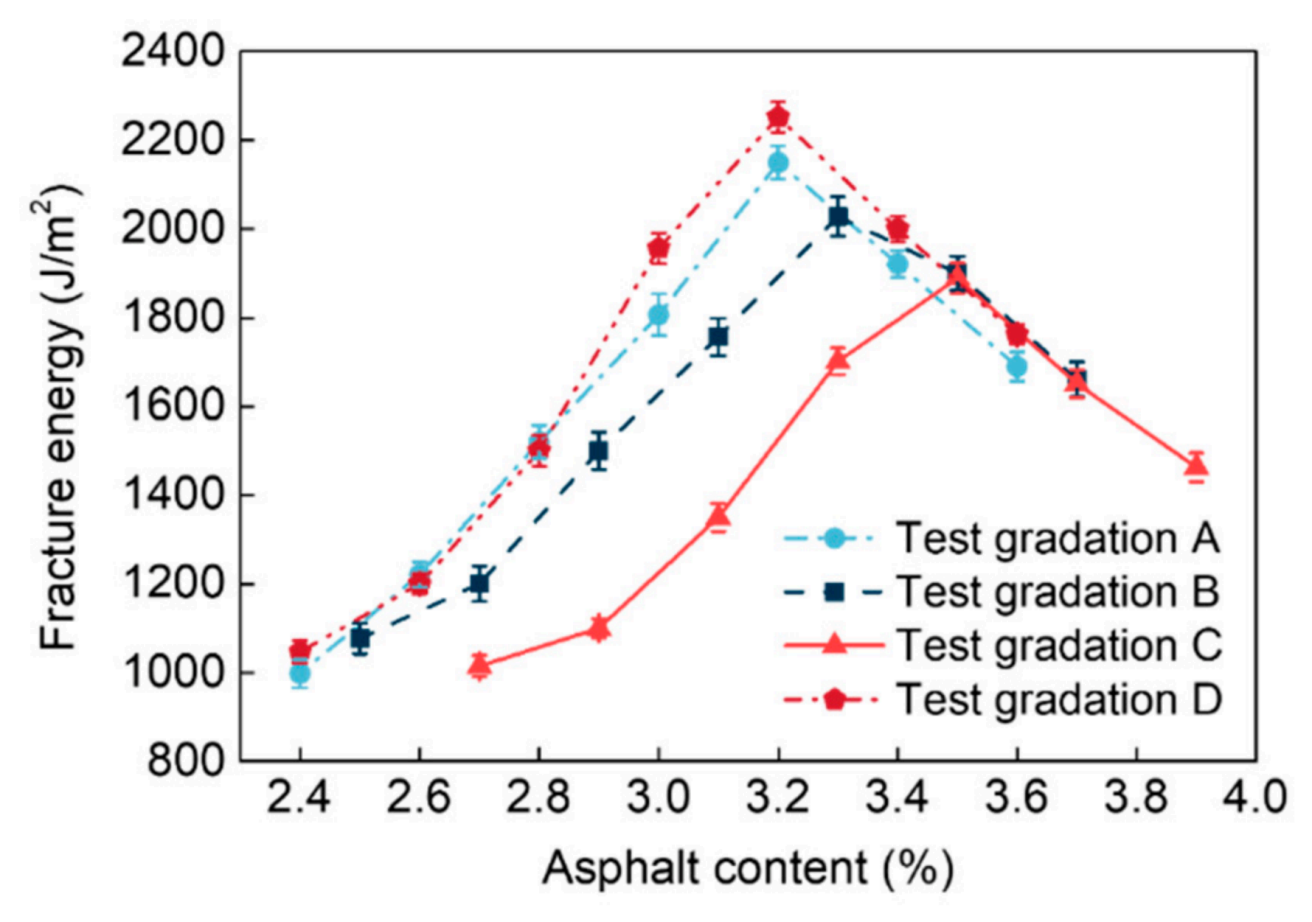
Curve of SLAM-50 fracture energy varying with asphalt content.

Figure 5 presents the fracture energy results of SLAM-50 with four aggregate gradations at different asphalt contents. As observed, the fracture energy exhibits a non-monotonic trend with increasing asphalt content, i.e., it increases initially and then decreases. The peak fracture energy values were obtained at asphalt contents of 3.2% for Gradation A (2149.7 J/m^2^), 3.3% for Gradation B (2028.1 J/m^2^), 3.5% for Gradation C (1888.9 J/m^2^), and 3.2% for Gradation D (2252.1 J/m^2^).

Gradations A and D generally exhibited higher fracture energy, whereas Gradation C consistently showed the lowest values. This difference is primarily associated with the characteristics of the aggregate skeleton. For Gradations A and D, the higher proportion of large-sized coarse aggregates together with a more continuous gradation promotes the development of a stable three-dimensional interlocked skeleton during compaction. Accordingly, crack propagation is more likely to be deflected by coarse aggregates, resulting in a more tortuous fracture path and greater energy dissipation. By contrast, Gradation C contains a lower coarse aggregate fraction, which leads to a less stable skeleton. In this case, cracking tends to propagate preferentially through the asphalt mastic and fine-aggregate matrix, producing a relatively straighter path and, consequently, lower fracture energy. This interpretation is consistent with the volumetric results in Section 4.1, where Gradations A and D exhibited lower *VV* and higher *VFA*, indicating more efficient aggregate packing and asphalt filling. Previous studies have also reported that aggregate skeleton structure and volumetric characteristics strongly influence cracking resistance and fracture behavior of asphalt mixtures [11,32].

### 4.4. Dynamic Stability Results

**Figure 6:**
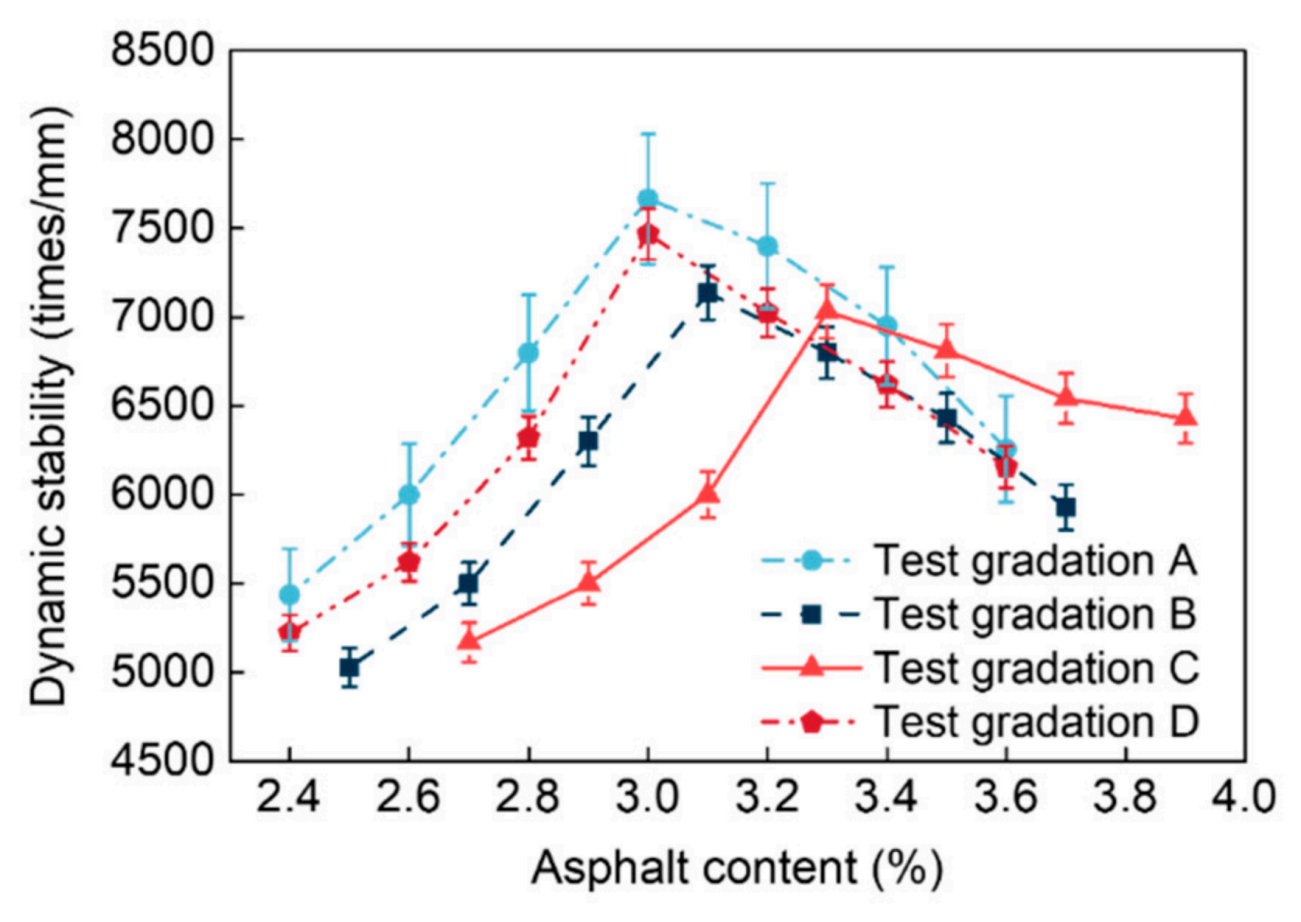
Curve of SLAM-50 dynamic stability varying with asphalt content.

Figure 6 illustrates the variation of dynamic stability with asphalt content. As shown, the dynamic stability of SLAM-50 exhibits a non-monotonic trend with increasing asphalt content, increasing initially and then decreasing after reaching a peak value. The maximum dynamic stability values were obtained at asphalt contents of 3.0% for Gradation A (7665.7 times/mm), 3.1% for Gradation B (7138.5 times/mm), 3.3% for Gradation C (7032.4 times/mm), and 3.0% for Gradation D (7468.3 times/mm).

A comparison among gradations indicates that Gradations A and D achieved relatively higher dynamic stability at their respective optimum asphalt contents. This suggests that gradations with a higher coarse aggregate fraction and a more stable load-bearing aggregate skeleton are beneficial for enhancing rutting resistance of asphalt mixtures. From a mechanistic perspective, the higher dynamic stability of Gradations A and D is mainly attributed to stronger stone-on-stone contact and lower air void contents, which reduce permanent deformation under repeated wheel loading. In contrast, Gradation C contains a higher fine aggregate proportion and a weaker coarse aggregate skeleton, making the mixture more dependent on asphalt mastic and therefore more susceptible to deformation. These observations are consistent with previous findings that *VMA*, *VFA*, and aggregate skeleton structure significantly affect rutting resistance [32].

### 4.5. Effect of Volume Parameters on SLAM-50 Performance Index

#### 4.5.1. Effect of *VV* on Performance

**Figure 7:**
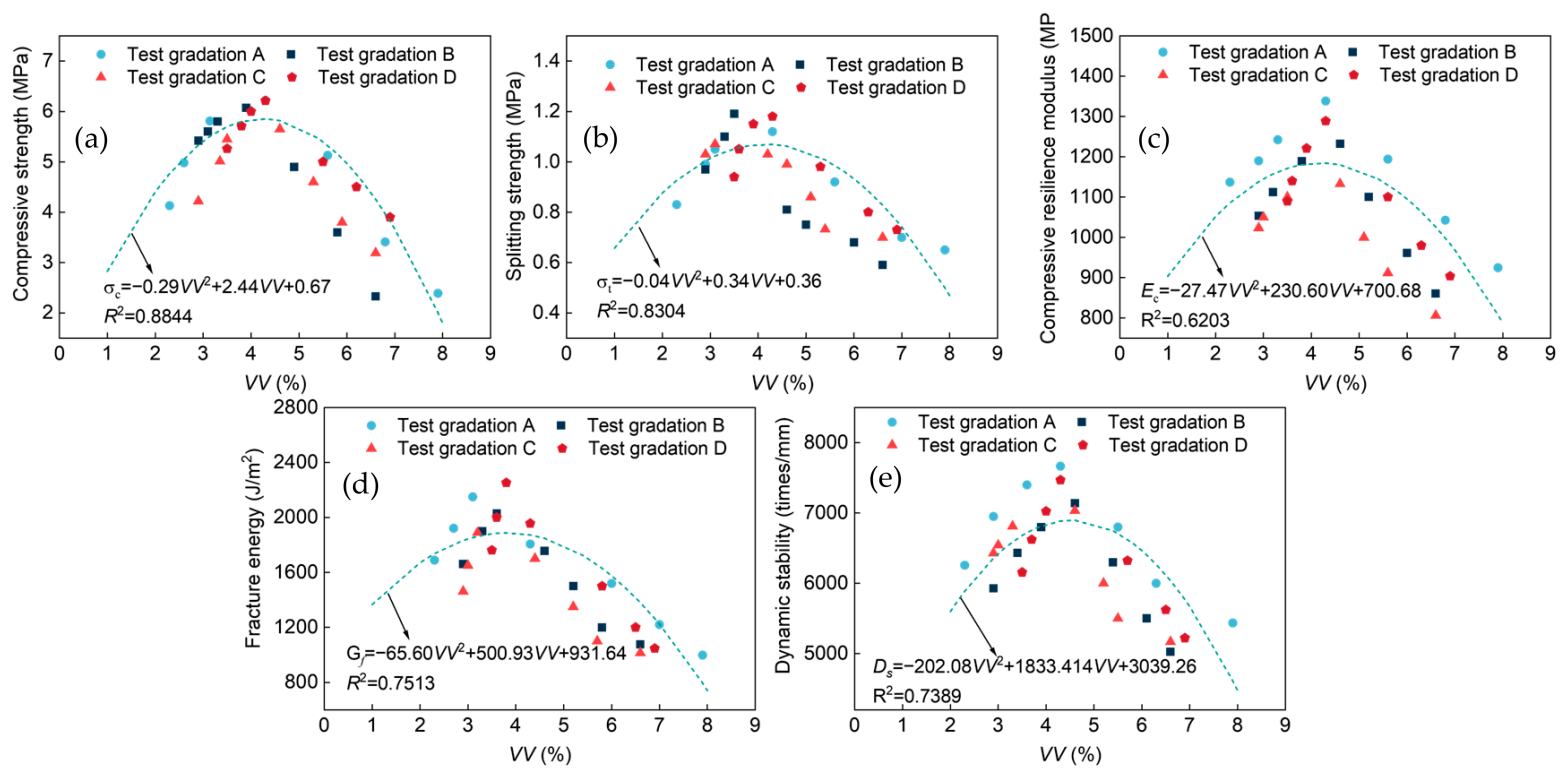
Relationship between *VV* and asphalt mixture performance: (**a**) Compressive strength ($\sigma_{c}$). (**b**) splitting strength ($\sigma_{sc}$). (**c**) Compressive resilience modulus ($E_{c}$). (**d**) Fracture energy ($G_{f}$). (**e**) Dynamic stability ($D_{s}$).

The above experimental results indicate that the relationships between volumetric parameters and the evaluated performance indices of SLAM-50 exhibit pronounced nonlinear characteristics. Figure 7 depicts the relationship between *VV* and the compressive strength ($\sigma_{c}$), splitting strength ($\sigma_{sc}$), compressive resilience modulus ($E_{c}$), fracture energy ($G_{f}$) and dynamic stability ($D_{s}$) for the four SLAM-50 gradations. For all gradations, $\sigma_{c}$, $\sigma_{sc}$, $E_{c}$, $G_{f}$ and $D_{s}$ initially increased and then decreased as *VV* grows. The performances of SLAM-50 asphalt mixtures reached their optimal levels when *VV* was approximately 4%. This phenomenon occurs because *VV* is inversely correlated with the asphalt content (as shown in Figure 3a). A higher VV corresponds to a lower asphalt content, which weakens the bonding between aggregates and results in reduced strength and cracking resistance. Conversely, an excessively low *VV* implies a relatively high amount of free asphalt, which may reduce stone-on-stone contact, weaken internal cohesion and frictional resistance within the aggregate skeleton, and consequently increase the susceptibility of the mixture to deformation. As a result, the overall mechanical performance of the asphalt mixture deteriorates when *VV* deviates significantly from its optimal range. To quantitatively describe the relationship between *VV* and the performance indices of SLAM-50, quadratic regression models were established based on the experimental data. As shown in Figure 7a–e, the coefficients of determination for $\sigma_{c}\sim VV$, $\sigma_{t}\sim VV$, $G_{f}\sim VV$ and $D_{s}\sim VV$ are 0.8844, 0.8304, 0.7513 and 0.7389, respectively, indicating relatively strong correlations. In contrast, the correlation between the compressive resilient modulus ($E_{c}$) and *VV* is comparatively weak, with an *R*^2^ value of only 0.6203. This weaker correlation may be attributed to the fact that mixture strength, low-temperature cracking resistance, and high-temperature rutting performance are primarily governed by the aggregate skeleton structure and asphalt–aggregate bonding characteristics. Meanwhile, the compressive resilient modulus is more sensitive to additional factors such as testing temperature, loading–unloading response, and rebound deformation measurement uncertainty, which may collectively reduce the goodness of fit between *VV* and $E_{c}$.

#### 4.5.2. Effect of *VFA* on Performance

**Figure 8:**
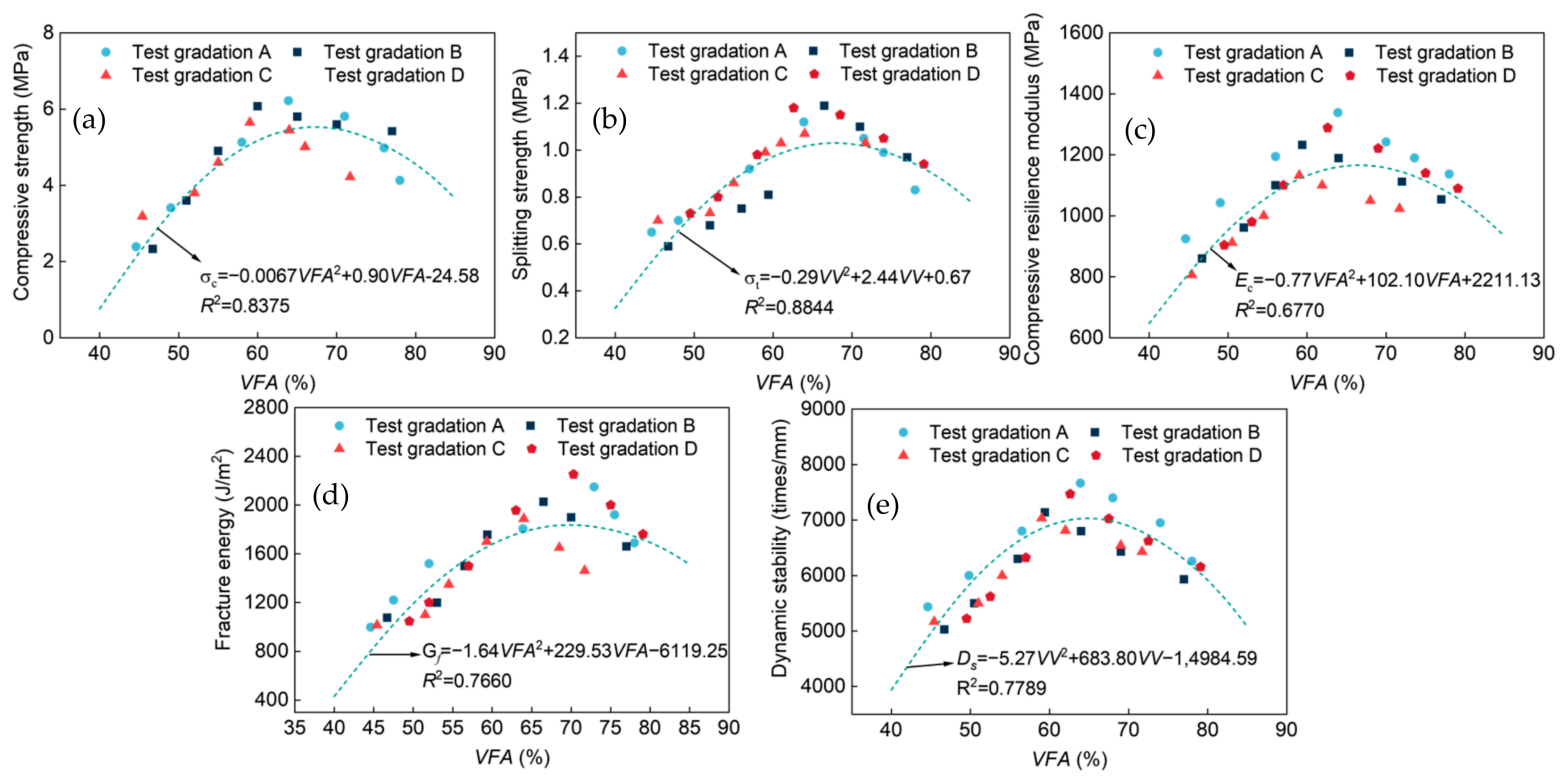
Relationship between *VFA* and mechanical strength: (**a**) Compressive strength. (**b**) Splitting strength. (**c**) Compressive resilience modulus ($E_{c}$). (**d**) Fracture energy ($G_{f}$). (**e**) Dynamic stability ($D_{s}$).

Figure 8 illustrates the relationships between the *VFA* and the compressive strength ($\sigma_{c}$), splitting strength ($\sigma_{sc}$), and compressive resilient modulus ($E_{c}$), fracture energy ($G_{f}$) and dynamic stability ($D_{s}$) of SLAM-50. Similar to the trends discussed in Section 4.5.1, all five performance indices exhibit a non-monotonic variation with increasing *VFA*, increasing initially and then decreasing. The best balance among performance indices is obtained when *VFA* is approximately 75%, in agreement with the observations in Section 4.5.1. This behavior can be attributed to the effect of asphalt content reflected by *VFA*. An increase in *VFA* generally corresponds to a higher asphalt content, which enhances internal cohesion and frictional resistance within the mixture, thereby improving its mechanical performance. However, with further increases in *VFA*, the presence of excessive free asphalt tends to reduce internal cohesion and friction resistance, leading to a decline in compressive strength, splitting strength, and compressive resilient modulus. Nonlinear regression analysis further indicates that *VFA* exhibits relatively strong correlations with compressive strength and splitting strength, with coefficients of determination (*R*^2^) of 0.8375 and 0.8844, respectively. In contrast, the correlation between *VFA* and the compressive resilient modulus is comparatively weak (*R*^2^ = 0.6770), which may be attributed to the fact that the resilient modulus is influenced by a broader range of factors beyond volumetric properties alone, such as temperature effects and loading–unloading response characteristics.

#### 4.5.3. Effect of *VMA* on Performance

**Figure 9:**
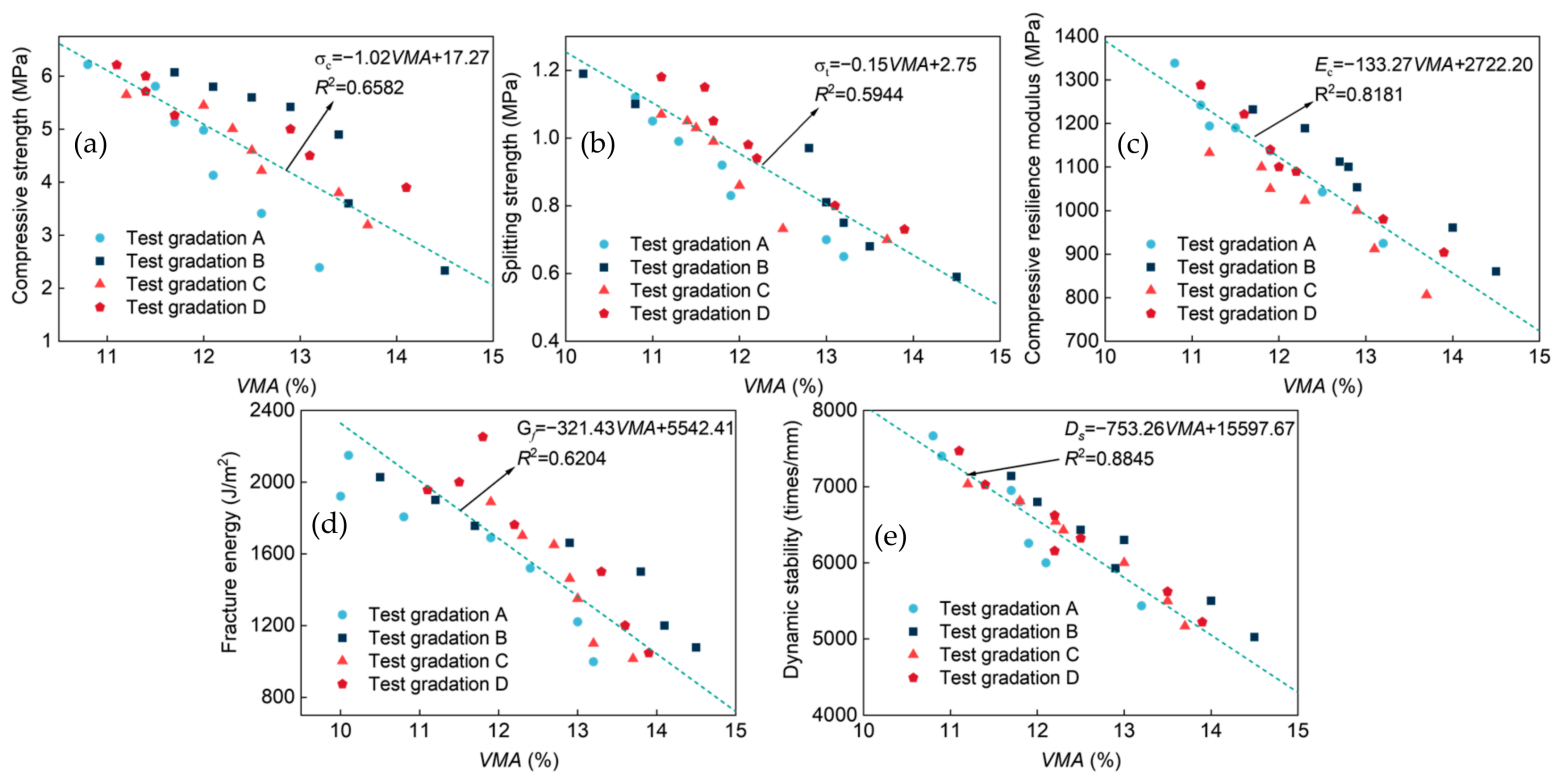
Relationship between *VMA* and asphalt mixture performance: (**a**) Compressive strength. (**b**) Splitting strength. (**c**) Compressive resilience modulus ($E_{c}$). (**d**) Fracture energy ($G_{f}$). (**e**) Dynamic stability ($D_{s}$).

Figure 9 presents the variations in SLAM-50 performance indices as a function of the *VMA*. As shown, the strength, high-temperature rutting resistance, and low-temperature cracking resistance of SLAM-50 decrease in an approximately linear manner with increasing *VMA*. This trend can be attributed to the fact that a higher *VMA* is generally associated with a looser aggregate skeleton and a lower degree of effective mastic filling at aggregate contacts, which weakens load transfer within the mixture and results in deteriorated performance.

Based on the experimental results, regression curves were established to quantify the relationships between *VMA* and the measured performance indices. It is observed that *VMA* exhibits relatively strong correlations with the indices $E_{c}$ and $D_{s}$ (as shown in Figure 9), indicating a higher goodness of fit. This can be explained by the sensitivity of these two indices to the stiffness of the aggregate skeleton and its resistance to shear deformation, whereas *VMA* is a primary volumetric parameter reflecting the compactness of the skeleton. Consequently, variations in *VMA* are more directly manifested in $E_{c}$ and $D_{s}$ compared with other performance measures.

The above experimental results indicate that the relationships between the volumetric parameters and performance indices of SLAM-50 exhibit pronounced nonlinear characteristics. Moreover, the observed variations are governed by the coupled effects of multiple factors, particularly aggregate gradation type and asphalt content. Under such multi-variable interactions, it is difficult to establish a unified and reliable performance prediction model using conventional regression-based fitting approaches alone. To further quantify the complex nonlinear mapping between volumetric parameters and mechanical performance under multi-parameter conditions, a BP–based artificial neural network (ANN) was therefore introduced in this study. The proposed framework was used to model the relationships between the *AC*, volumetric parameters, density of SLAM-50 ($\rho_{f}$) and GT and performance indices of SLAM-50 and to enable a systematic comparative analysis of prediction capability.

## 5. BP Neural-Network-Based Performance Prediction Model for SLAM-50 Under Different Volumetric Conditions

### 5.1. Determination of Model Parameters

Based on the above experiments, the input layer of the BP neural network was defined using six variables: *AC*, *VV*, *VFA*, *VMA*, density of SLAM-50 ($\rho_{f}$) measured during the experiment and GT. Set the gradation type input parameter to 1–4, corresponding to gradations A–D, respectively. The output layer consisted of five SLAM-50 performance indicators, namely compressive strength, splitting strength, compressive resilient modulus, fracture energy, and dynamic stability. The BP neural network was trained and validated using the experimental dataset obtained under different volume parameters, covering mechanical performance, low-temperature cracking resistance, and high-temperature rutting resistance of SLAM-50. Prior to training, all input and output variables were normalized to the range of [0, 1] using the min–max scaling method (Equation (12)) to mitigate the influence of differing magnitudes and units. The normalized dataset was then randomly divided into training, validation, and testing subsets to develop the model and evaluate its generalization performance. The ranges of all input and output variables are summarized in Table 7.

### 5.2. Pearson Correlation Analysis

**Figure 10:**
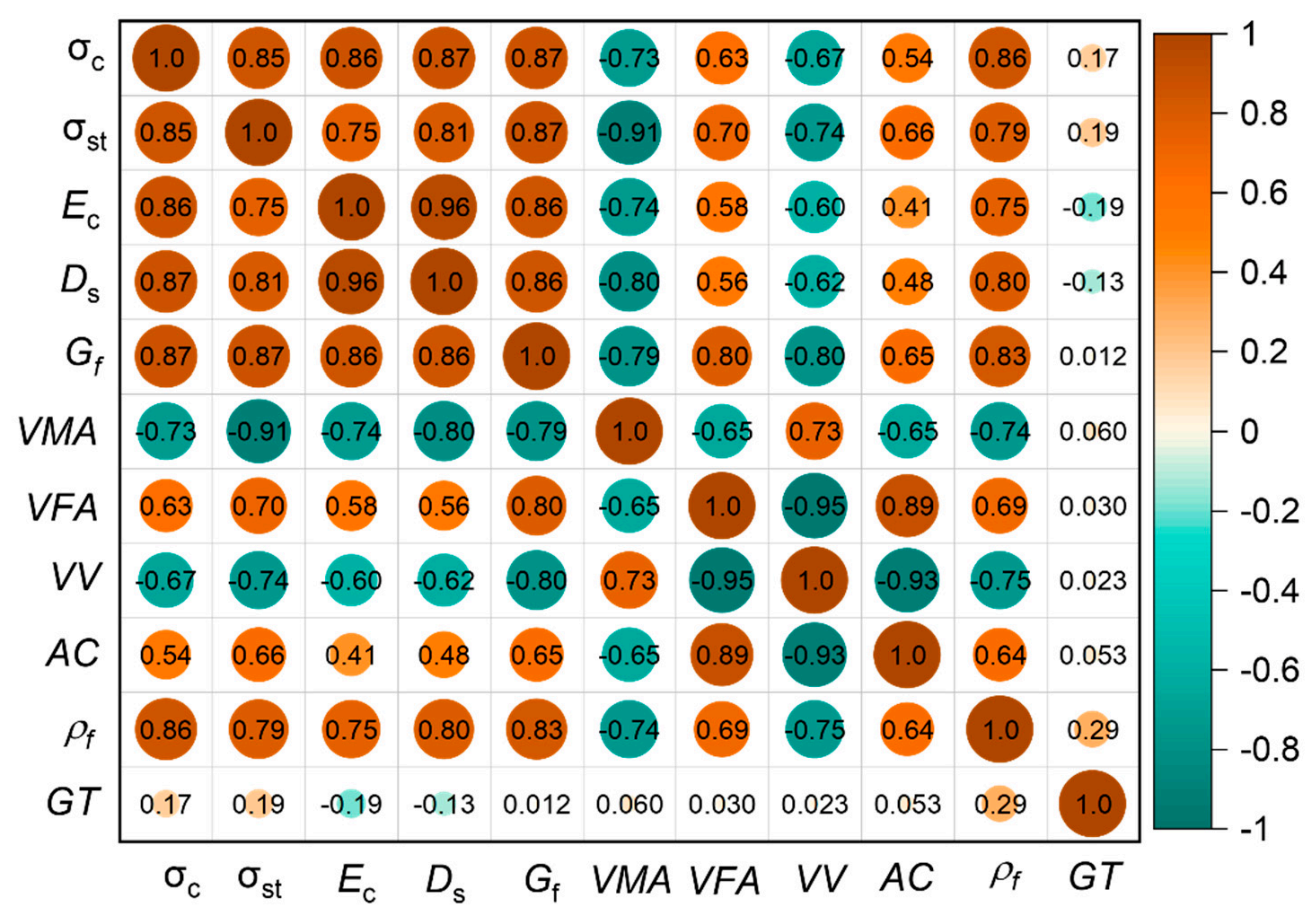
Pearson correlation heat map.

A cross-correlation analysis was performed on the input and output parameters defined in Section 5.1, and the results are shown in Figure 10. It can be observed that compressive strength ($\sigma_{c}$), splitting tensile strength ($\sigma_{sc}$), resilient modulus ($E_{c}$), dynamic stability ($D_{s}$), and fracture energy ($G_{f}$) were all strongly and positively correlated with each other (r = 0.75~0.96), indicating that mixtures with higher load-bearing capacity generally also exhibited better stiffness, rutting resistance, and fracture resistance. In contrast, *VMA* and *VV* showed predominantly negative correlations with these performance indicators. *VMA* exhibited strong negative correlations with $\sigma_{c}$, $\sigma_{sc}$, $E_{c}$, $G_{f}$ and $D_{s}$ (r = −0.73~mechanical performance −0.91), while *VV* was also negatively correlated with these performance-related variables (r = −0.60~−0.80), suggesting that excessive internal void-related characteristics were generally detrimental to the overall mechanical performance of the mixtures. By comparison, *VFA*, *AC*, and density of SLAM-50 ($\rho_{f}$) were positively associated with most mechanical properties. *VFA* showed moderate-to-strong positive correlations with $\sigma_{c}$, $\sigma_{sc}$, $E_{c}$, $G_{f}$ and $D_{s}$ (r = 0.56~0.80), and *AC* also displayed positive correlations with these indices (r = 0.41~0.66), implying that, within the investigated range, higher effective asphalt filling and asphalt content contributed to improved mechanical response. $\rho_{f}$ was positively correlated with all major mechanical properties (r = 0.75~0.86), further highlighting the importance of mixture compactness. Among the volumetric variables, the strongest relationships were found between *VFA* and *VV* (r = −0.95), *AC* and *VV* (r = −0.93), and *AC* and *VFA* (r = 0.89), which reflects the intrinsic interdependence among binder content, void structure, and aggregate packing state. In contrast, GT exhibited only weak correlations with most continuous variables (|r| ≤ 0.29). Overall, the correlation pattern suggests that the structural integrity and mechanical performance of super-large aggregate asphalt mixtures are governed primarily by the coupled effects of void structure, asphalt filling condition and gradation type alone.

### 5.3. Determination of Hidden Layer Nodes

For a BP neural network, the number of neurons in the input and output layers is determined by the dimensions of the input and output variables, whereas the optimal number of neurons in the hidden layers remains uncertain. To date, no unified theoretical framework exists for determining the number of hidden-layer neurons, and most studies rely on empirical or trial-and-error approaches. In this study, the number of hidden-layer nodes was designed by considering both the scale of the experimental dataset and the complexity required to accurately capture the nonlinear relationships within the model. The specific configurations evaluated are illustrated in Table 7. To identify a suitable network structure, the training convergence behavior, model complexity, and prediction stability were considered. For each hidden-layer configuration, the network was trained independently ten times, and the averaged training RMSE values were used to reduce the influence of random weight initialization. The testing set was not used for network-structure selection and was only employed to evaluate the generalization performance of the final model.

**Figure 11:**
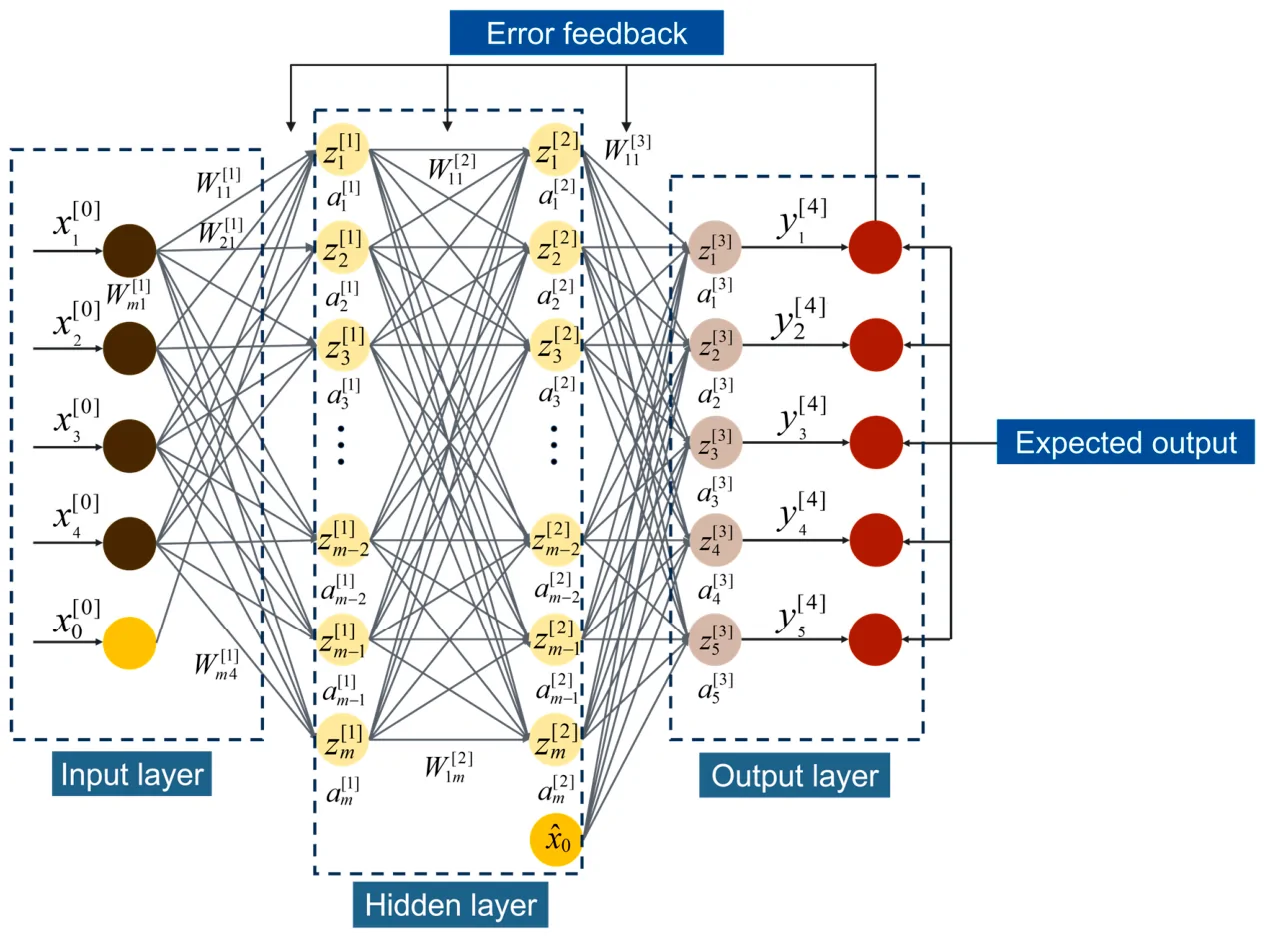
BP neural network structure diagram [48].

Table 8 summarizes the error analysis results for different neural network node configurations. The results indicate that the prediction accuracy varies significantly with the number of hidden-layer nodes. When the hidden-layer structure was set to 256 + 256 nodes, the RMSE values of the five output parameters on the training set were 0.662, 0.1361, 216.56, 401.18, and 885.67, respectively, while the corresponding RMSE values on the test set were 0.683, 0.156, 225.78, 372.82, and 794.27. Under this configuration, both the training and test errors were relatively small and well balanced, indicating good generalization performance. Accordingly, the BP neural network adopted in this study consists of two hidden layers, each containing 256 neurons. The final architecture of the neural network is shown in Figure 11.

### 5.4. Training and Convergence Analysis of Neural Network Model

**Figure 12:**
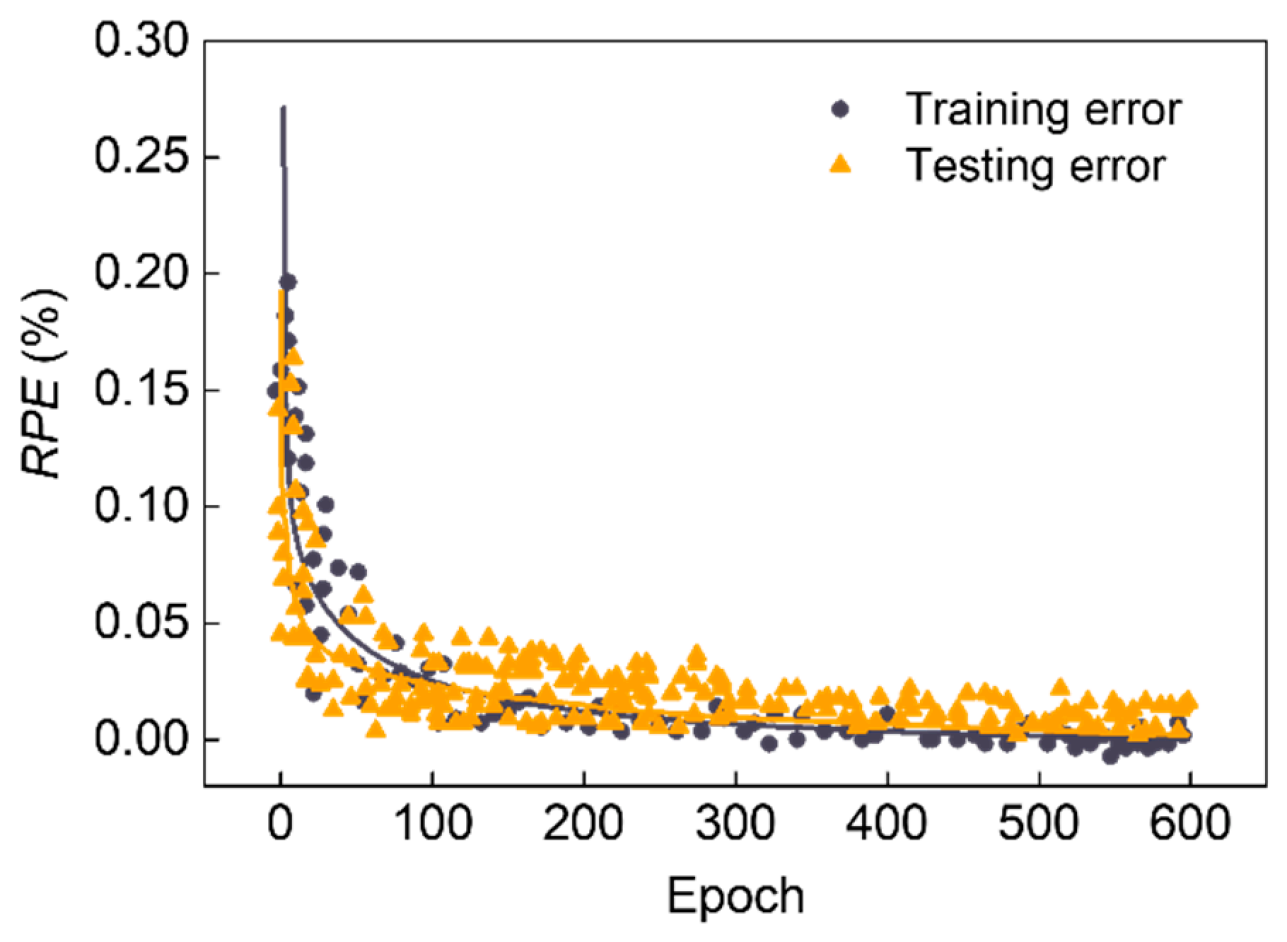
Error variation during training and testing.

This section develops a BP neural network model in a Python 3.11 environment to predict asphalt mixture performance using volumetric parameters and asphalt content. First, all variables were normalized prior to modeling. To mitigate the risk of overfitting due to the limited dataset, all input and output variables were normalized to [0, 1]. The 84 samples were divided into a training set (*n* = 59) and an independent testing set (*n* = 25). The testing set was not used during model training and was only employed to evaluate the generalization performance of the trained model. The network training process was monitored by comparing the evolution of training and testing errors. The BP neural network was trained using the Adam optimizer. Several candidate initial learning rates, including 0.0005, 0.001, 0.003, 0.005, and 0.01, were evaluated based on convergence behavior and prediction performance. Lower learning rates resulted in slower convergence, whereas higher learning rates produced larger fluctuations during training. Therefore, an initial learning rate of 0.003 was adopted to balance convergence speed and training stability. Figure 12 shows the evolution of model error during the training and testing stages. The training error gradually stabilized after 140 iterations, reaching a minimum of 1.081%, whereas the testing error converged after approximately 270 iterations with a minimum value of 1.016%. The relatively close convergence of training and testing errors indicates that no obvious divergence between model fitting and generalization performance was observed within the investigated dataset. Therefore, to balance predictive performance and computational cost, the number of training iterations (epochs) was set to 270 for the proposed neural-network model.

### 5.5. Performance Prediction Results

**Figure 13:**
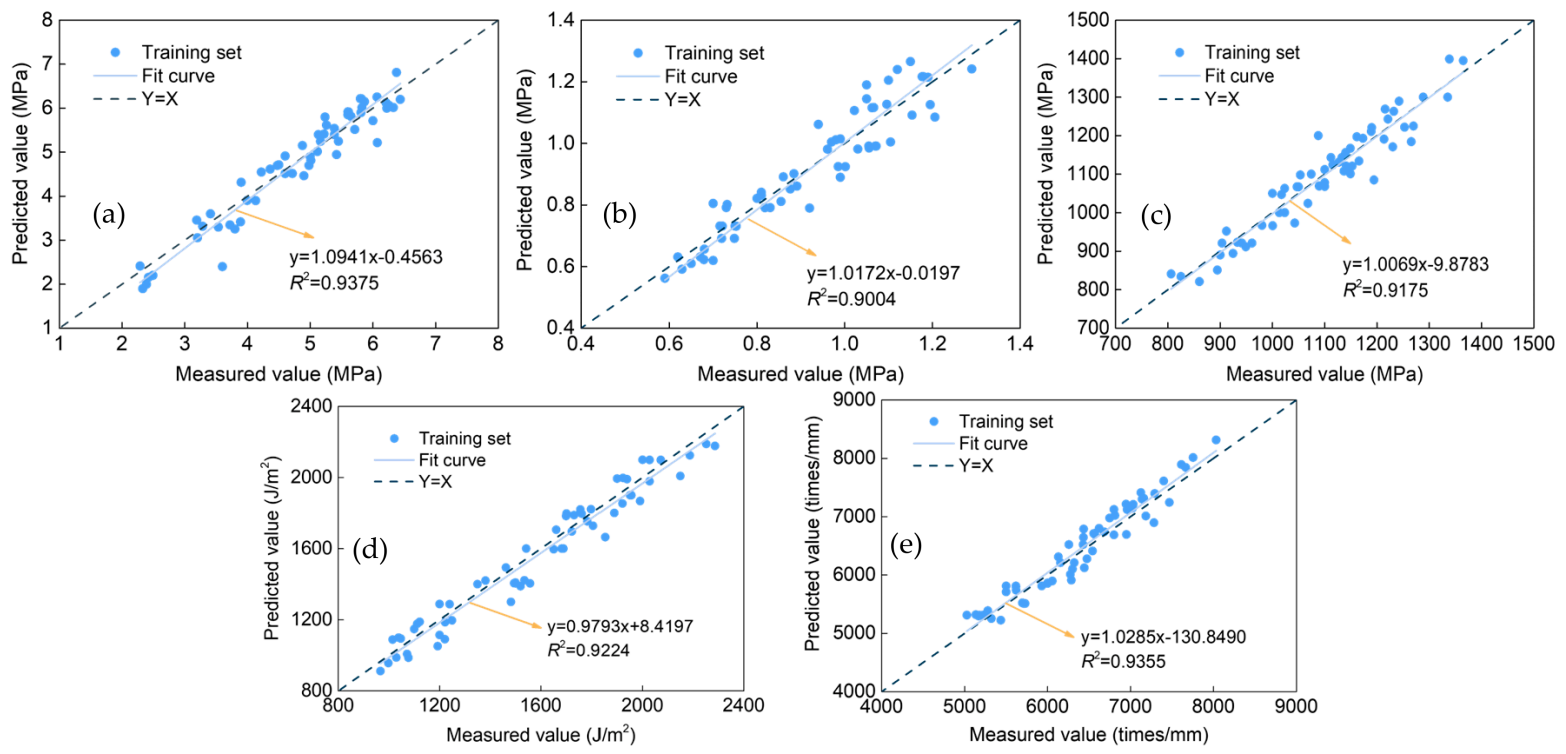
The correlation between predicted values and measured values in the training set: (**a**) Compressive strength. (**b**) Splitting strength. (**c**) Compressive resilience modulus ($E_{c}$). (**d**) Fracture energy ($G_{f}$). (**e**) Dynamic stability ($D_{s}$).

The prediction results of the optimized BP neural network for the training set are presented in Figure 13, and the corresponding prediction errors are summarized in Table 9. As shown, the measured and predicted values exhibit strong agreement, with most data points distributed closely around the 1:1 reference line (Y = X). Moreover, Table 9 indicates that the coefficients of determination (*R*^2^) for all five performance indices are higher than 0.91, while the mean absolute error (MAE) and root mean square error (RMSE) remain low. These findings demonstrate that the proposed model can effectively capture the relationships between volumetric parameters and performance indices.

**Figure 14:**
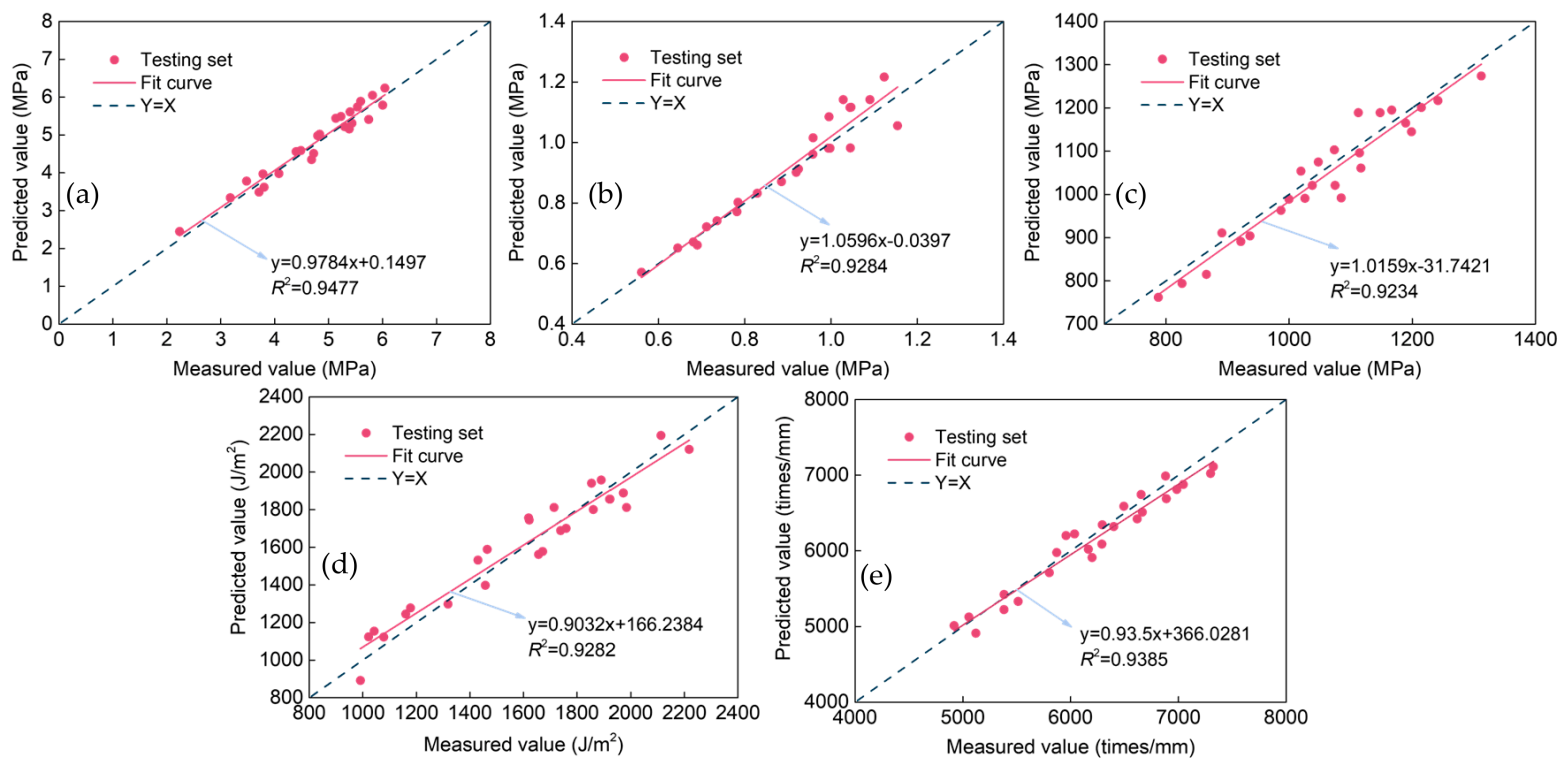
The correlation between predicted values and measured values in the testing set: (**a**) Compressive strength. (**b**) Splitting strength. (**c**) Compressive resilience modulus ($E_{c}$). (**d**) Fracture energy ($G_{f}$). (**e**) Dynamic stability ($D_{s}$).

Figure 14 further presents the model predictions for the testing set, and the corresponding error statistics are summarized in Table 10. A strong agreement between predicted and measured values is observed for all five performance indices, with correlation coefficients of 0.9477, 0.9284, 0.9234, 0.9282, and 0.9385, respectively, confirming the high predictive accuracy of the model. Moreover, the low MAE and RMSE values in Table 10 further demonstrate the robustness and effectiveness of the proposed approach. Taken together, these results indicate that the optimized BP neural network can accurately predict the key performance indicators of SLAM-50 using volumetric parameters as inputs.

## 6. Conclusions

This study investigated SLAM-50 to elucidate the relationships among aggregate gradation, asphalt content, volumetric properties, and key performance indicators, including strength, low-temperature cracking resistance, and high-temperature stability. The correlations between volumetric parameters and performance indices were further analyzed. Finally, a BP neural-network-based prediction model was developed to evaluate the primary performance of SLAM-50. The main conclusions are summarized as follows:

(1) The effects of asphalt content on the volumetric properties and performance indices of SLAM-50 were examined. With increasing asphalt content, *VV* decreased, *VMA* decreased initially and then increased, and *VFA* increased monotonically. All performance indices exhibited a similar non-monotonic trend, increasing first and then decreasing. The recommended asphalt content for achieving a balanced performance is 3.0–3.2%.
(2) Regression relationships between volumetric parameters and performance indices were established. The results show that, as *VV* and *VFA* increased, all performance indices followed a convex parabolic trend (i.e., increased initially and then decreased), whereas the performance indices varied approximately linearly with *VMA*. The correlation coefficients between *VV* and the performance indices ranged from 0.6203 to 0.8844; those between *VFA* and the performance indices ranged from 0.6770 to 0.8844; and those between *VMA* and the performance indices ranged from 0.5944 to 0.8845. These results indicate that linear regression provides useful insight into trends for individual performance indicators, but it may not fully capture the multi-input–multi-output and nonlinear interactions observed in SLAM-50 mixtures. The BP neural network was therefore adopted to model these coupled relationships and demonstrated high predictive accuracy (R^2^ > 0.91 for all outputs).
(3) Three volumetric parameters (*VV*, *VFA*, and *VMA*), asphalt content, density of SLAM-50, and gradation type were selected as input variables, while five performance indices (compressive strength, splitting strength, compressive resilient modulus, fracture energy, and dynamic stability) were used as outputs to develop a BP neural network model. The developed model achieved R^2^ values greater than 0.91 for both the training and testing datasets, indicating that the proposed framework can effectively characterize the nonlinear and coupled relationships among mixture design variables and performance responses of SLAM-50.

In summary, this study establishes quantitative relationships between volumetric parameters and multiple performance indicators of SLAM-50 and demonstrates the feasibility of predicting mixture performance using a BP neural-network framework. The proposed approach provides a data-driven reference for performance-oriented design and optimization of super-large aggregate asphalt mixtures. The present study focused on SLAM-50 mixtures and evaluated compressive strength, splitting strength, compressive resilient modulus, fracture energy, and dynamic stability. Fatigue performance, moisture susceptibility, aging durability, and applicability to other asphalt mixture types were beyond the scope of this work. In future studies, expanded datasets will be used to include additional performance indicators, compare different machine-learning algorithms, and conduct comprehensive statistical analyses, including ANOVA, multiple comparison tests, and cross-validation, to further improve model robustness and validate the significance of observed differences among gradations. While economic and environmental analyses were not conducted in this study, the proposed predictive framework provides a basis for future evaluation of the cost-effectiveness, sustainability, and potential environmental impacts of SLAM-50 mixtures.

## Figures and Tables

**Table 1:** Karamay 90# matrix asphalt index.

Test Projects		Unit	Specified Value	Test Result	Test Method [10]
Needle penetration (25 °C, 100 g, 5 s)		0.1 mm	80~100	89	T0604-2011
Penetration index (PI)		−1.2	−1.5~+1.0	−1.2	T0604-2011
Ductility (10 °C)		cm	≥45	>100	T0605-2011
Softening point (R&B), °C		°C	≥45	45.5	T0606-2011
Dynamic viscosity (60 °C)		Pa·s	≥160	276.3	T0620-2000
Wax content		%	≤2.2	2	T0615-2011
Flash point		°C	≥245	314	T0611-2011
Solubility (trichloroethylene)		%	≥99.5	99.76	T0607-2011
Density (15 °C)		g/cm^3^	-	0.981	T0603-2011
Rotary film oven test (163 °C)	Mass change	%	±0.8	−0.047	T0609-2011

**Table 2:** Technical indicators of coarse aggregates.

Test Projects	Unit	Specified Value	Test Result	Test Method [44]
Stone crushing value	%	≤26	12.7	T0316-2005
Apparent relative density	-	≥2.6	2.818	T0304-2005
Los Angeles wear loss	%	≤28	18.6	T0317-2005
Needle flake particle content	%	≤15	7.3	T0312-2005
Moisture content	%	≤2.0	1.432	T0304-2005
Solidity	%	≤12	3.2	T0314-2000
Slime content	%	≤3	1.7	T0320-2000

**Table 3:** Technical indicators of fine aggregates.

Test Index	Unit	Specified Value	Test Result	Test Method [44]
Apparent relative density	-	≥2.5	2.802	T0328-2005
Sand equivalent	%	≥50	64	T0344-2005
Angularity (flow time)	s	≥30	39.7	T0345-2005

**Table 4:** Technical indicators of the mineral powder.

Test Index		Unit	Specified Value	Test Result	Test Method [44]
Apparent relative density		-	≥2.5	2.727	T0352-2000
Screen analysis	<0.6 mm	%	100	100	T0344-2005
	<0.15 mm		91.2	90~100	
	<0.075 mm		76.4	75~100	
Hydrophilic coefficient		-	<1	0.73	T0353-2000

**Table 5:** Aggregate gradation compositions of Gradations A–D designed for SLAM-50.

Gradation Type	Passing Percentage (%)										
	53	37.5	19	9.5	4.75	2.36	1.18	0.6	0.3	0.15	0.075
A	100	65	55	40	35	26.4	20.7	16.6	11.6	8.6	4.3
B	100	70	60	43	33	24.8	19.4	15.5	10.7	7.9	3.8
C	100	75	65	46	35	26.5	20.9	16.9	12	9	4.7
D	100	65	55	40	30.5	22.8	17.8	14.2	9.7	7.1	3.4

**Table 6:** Asphalt content of each gradation in the experiment.

Gradation	Asphalt Content (%)
A	2.4, 2.6, 2.8, 3.0, 3.2, 3.4, 3.6
B	2.5, 2.7, 2.9, 3.1, 3.3, 3.5, 3.7
C	2.7, 2.9, 3.1, 3.3, 3.5, 3.7, 3.9
D	2.4, 2.6, 2.8, 3.0, 3.2, 3.4, 3.6

**Table 7:** Input and output parameters of BP neural network model.

Model Properties		Unit	Range of Values	
			Max	Min
Input parameters	*AC*	%	3.6	2.4
	*VV*	%	7.9	2.9
	*VFA*	%	79.1	47.5
	*VMA*	%	14.5	11
	$\rho_{f}$	g/cm^3^	2.43	2.52
	*GT*	/	4	1
Output parameters	Compressive strength	MPa	6.00	2.33
	Splitting strength	MPa	1.12	0.59
	Compressive resilience modulus	MPa	1338.5	912
	Fracture energy	J/m^2^	2252.1	1077.1
	Dynamic stability	times/mm	7138.5	5026.9

**Table 8:** Error analysis of different neural network nodes.

Input Layer	Hidden Layer		Output Layer	Prediction Index	Training RMSE	Test RMSE
	One	Two				
4	64	32	5	Compressive strength (MPa)	1.079	1.027
				Splitting strength (MPa)	0.212	0.230
				Compressive resilience modulus (MPa)	318.8	301.0
				Fracture energy (J/m^2^)	582.8	532.3
				Dynamic stability (times/mm)	1205.6	1307.2
4	64	64	5	Compressive strength (MPa)	1.019	1.023
				Splitting strength (MPa)	0.206	0.225
				Compressive resilience modulus (MPa)	312.09	285.52
				Fracture energy (J/m^2^)	475.91	499.26
				Dynamic stability (times/mm)	1117.80	1163.41
4	128	32	5	Compressive strength (MPa)	1.044	0.965
				Splitting strength (MPa)	0.1817	0.1283
				Compressive resilience modulus (MPa)	332.651	180.056
				Fracture energy (J/m^2^)	478.84	413.31
				Dynamic stability (times/mm)	1170.8	1021.56
4	128	64	5	Compressive strength (MPa)	0.8903	0.7981
				Splitting strength (MPa)	0.1754	0.1359
				Compressive resilience modulus (MPa)	263.97	246.08
				Fracture energy (J/m^2^)	512.52	459.27
				Dynamic stability (times/mm)	1063.86	921.26
4	128	128	5	Compressive strength (MPa)	0.8288	0.8217
				Splitting strength (MPa)	0.1641	0.1430
				Compressive resilience modulus (MPa)	252.33	242.91
				Fracture energy (J/m^2^)	419.81	420.12
				Dynamic stability (times/mm)	891.42	821.96
4	256	64	5	Compressive strength (MPa)	1.012	0.7988
				Splitting strength (MPa)	0.1957	0.1313
				Compressive resilience modulus (MPa)	307.39	235.46
				Fracture energy (J/m^2^)	539.30	478.05
				Dynamic stability (times/mm)	1125.14	1003.75
4	256	128	5	Compressive strength (MPa)	0.8423	0.7567
				Splitting strength (MPa)	0.1601	0.1399
				Compressive resilience modulus (MPa)	241.10	250.31
				Fracture energy (J/m^2^)	446.67	441.99
				Dynamic stability (times/mm)	901.88	795.21
4	256	256	5	Compressive strength (MPa)	0.662	0.683
				Splitting strength (MPa)	0.1361	0.156
				Compressive resilience modulus (MPa)	216.56	225.78
				Fracture energy (J/m^2^)	401.18	372.82
				Dynamic stability (times/mm)	885.67	794.27
4	512	128	5	Compressive strength (MPa)	0.8469	0.7523
				Splitting strength (MPa)	0.1998	0.1340
				Compressive resilience modulus (MPa)	316.25	204.62
				Fracture energy (J/m^2^)	525.78	595.19
				Dynamic stability (times/mm)	656.48	741.16
4	512	256	5	Compressive strength (MPa)	1.258	1.272
				Splitting strength (MPa)	0.2610	0.2576
				Compressive resilience modulus (MPa)	401.63	424.30
				Fracture energy (J/m^2^)	816.04	825.73
				Dynamic stability (times/mm)	1019.63	1154.23
4	512	512	5	Compressive strength (MPa)	4.2686	4.2966
				Splitting strength (MPa)	0.8677	0.8426
				Compressive resilience modulus (MPa)	1414.22	1254.3
				Fracture energy (J/m^2^)	2589.3	2701.6
				Dynamic stability (times/mm)	2493.56	2126.43

**Table 9:** Evaluation of BP neural network model’s predictive ability in training set.

Performance Index	Unit	*R* ^2^	MAE	RMSE
Compressive strength	MPa	0.9375	0.3865	0.5166
Splitting strength	MPa	0.9004	0.1321	0.1749
Compressive resilience modulus	MPa	0.9175	142.6168	177.2026
Fracture energy	J/mm^2^	0.9224	153.5110	201.7657
Dynamic stability	Times/mm	0.9355	271.0326	368.3812

**Table 10:** Evaluation of BP neural network model’s predictive ability in testing set.

Performance Index	Unit	*R* ^2^	MAE	RMSE
Compressive strength	MPa	0.9477	0.3431	0.4457
Splitting strength	MPa	0.9284	0.1160	0.1378
Compressive resilience modulus	MPa	0.9234	129.0698	155.4713
Fracture energy	J/mm^2^	0.9282	198.6937	241.3974
Dynamic stability	Times/mm	0.9385	269.6226	328.1459

## Data Availability

The original contributions presented in this study are included in the article. Further inquiries can be directed to the corresponding authors.
